# The efficacy of a task model approach to ADL rehabilitation in stroke apraxia and action disorganisation syndrome: A randomised controlled trial

**DOI:** 10.1371/journal.pone.0264678

**Published:** 2022-03-03

**Authors:** Jo Howe, Winnie Chua, Emily Sumner, Bogna Drozdowska, Rosanna Laverick, Rachel L. Bevins, Emilie Jean-Baptiste, Martin Russell, Pia Rotshtein, Alan M. Wing

**Affiliations:** 1 School of Psychology, College of Life and Environmental Sciences, University of Birmingham, Birmingham, United Kingdom; 2 School of Pharmacy, Aston University, Birmingham, United Kingdom; 3 Institute of Cardiovascular Sciences, College of Medical and Dental Sciences, University of Birmingham, Birmingham, United Kingdom; 4 School of Life Sciences, Faculty of Health and Life Sciences, Coventry University, Coventry, United Kingdom; 5 School of Electronic, Electrical and Systems Engineering, College of Engineering and Physical Sciences, University of Birmingham, Birmingham, United Kingdom; Prince Sattam Bin Abdulaziz University, College of Applied Medical Sciences, SAUDI ARABIA

## Abstract

**Background:**

Apraxia and action disorganization syndrome (AADS) after stroke can disrupt activities of daily living (ADL). Occupational therapy has been effective in improving ADL performance, however, inclusion of multiple tasks means it is unclear which therapy elements contribute to improvement. We evaluated the efficacy of a task model approach to ADL rehabilitation, comparing training in making a cup of tea with a stepping training control condition.

**Methods:**

Of the 29 stroke survivors with AADS who participated in this cross-over randomized controlled feasibility trial, 25 were included in analysis [44% females; mean(SD) age = 71.1(7.8) years; years post-stroke = 4.6(3.3)]. Participants attended five 1-hour weekly tea making training sessions in which progress was monitored and feedback given using a computer-based system which implemented a Markov Decision Process (MDP) task model. In a control condition, participants received five 1-hour weekly stepping sessions.

**Results:**

Compared to stepping training, tea making training reduced errors across 4 different tea types. The time taken to make a cup of tea was reduced so the improvement in accuracy was not due to a speed-accuracy trade-off. No improvement linked to tea making training was evident in a complex tea preparation task (making two different cups of tea simultaneously), indicating a lack of generalisation in the training.

**Conclusions:**

The clearly specified but flexible training protocol, together with information on the distribution of errors, provide pointers for further refinement of task model approaches to ADL rehabilitation. It is recommended that the approach be tested under errorless learning conditions with more impaired patients in future research.

**Trial registration:**

Retrospectively registered at ClinicalTrials.gov on 5^th^ August 2019 [NCT04044911] https://clinicaltrials.gov/ct2/show/NCT04044911?term=Cogwatch&rank=1

## Background

Activities of daily living (ADLs) after stroke are often impaired by hemiparesis resulting in weak, slow and uncoordinated movements [1] but also by cognitive disturbances of action. Such cognitive disturbances include apraxia [2] and action disorganization syndrome [3], which affect the planning and sequencing of action. Apraxia is defined as deficits in conceptual representation of actions and of tool use (e.g. problems miming use for hammer), whereas action disorganisation syndrome is defined as disordered elements of action sequence. The left inferior parietal lobe has often been implicated in limb apraxia [4] as it plays a role in action planning and prediction, motor imagery and anticipatory control of movement, as well as in predicting the movements of others, therefore, left-hemisphere stroke is a major source of action errors in apraxia [5]. However, apraxia deficits have also been observed in patients with right hemisphere damage, involving specific spatial and temporal errors primarily when performing transitive gestures, in contrast with left hemisphere damaged patients who produced a wide range of spatiotemporal and conceptual errors for both transitive and intransitive gestures, when measured using the Florida Apraxia Battery [6]. Similarly, both left and right hemisphere stroke patients showed worse performance on the FAST-R and on transitive movement scores, although left hemisphere patients showed more content errors [7].

For post-stroke patients, neurorehabilitation is usually delivered by a multidisciplinary team comprising a core group of occupational therapists, physiotherapists, speech and language therapists and nurses. Patient goals for neurorehabilitation following stroke are generally functional in nature, personalised to the needs of the individual and frequently focus on ADLs. The rehabilitation of ADLs after stroke is commonly delivered by occupational therapists while physiotherapists generally focus on improving hemiparetic control of movement [8, 9]. The inability to successfully complete ADLs can significantly impact upon the stroke survivor’s ability to live independently as well as reintegrate back into the community and is influenced by the presence of physical or cognitive impairments. Despite the importance of being able to successfully complete ADLs, research investigating ADL performance following stroke is limited [10–12] and it is currently unknown which is the most effective way to target the rehabilitation of ADLs in stroke survivors although recent randomised-control trials for upper limb apraxia have shown promise [13]. Rehabilitation of impairments in ADLs associated with apraxia and action disorganization syndrome (AADS) after stroke is the focus of the present paper.

Occupational therapy (OT) for stroke typically comprises: assessment, goal setting, training of ADLs, transfers and mobility, and environmental adaptations [14]. The ADLs may include a range of self-care activities such as dressing, washing, using the toilet, as well as food and drink preparation and household chores. A Cochrane review [15] of randomized controlled trials (RCTs) has shown OT to be effective for stroke patients, although a failure to demonstrate benefit of OT for stroke patients has been noted in care home settings [14]. Such RCTs of OT usually involve training that is tailored to individual patient goals, with the specific ADL and learning conditions varying across patients. In consequence, the content of treatment is not fixed across patients, which makes it difficult to determine which task elements contribute to improvement.

An exception to the task varying approach to ADL training was taken by Walker et al. [16] who focused on a single task, dressing. In a randomized control design, they compared conventional OT in one group with OT supplemented by neuropsychological assessment in another group. In both groups dressing improved compared to baseline. The difference between the two groups was not statistically reliable. However, there was a trend towards improved dressing for right hemisphere stroke patients who received the neuropsychological approach which included elements such as cueing and alerting. Moreover, significant gains in spatial attention in those receiving the neuropsychological approach were noted which could be advantageous in subsequent training of other ADLs.

In OT, training a specific ADL commonly involves first carrying out an activity analysis in order to specify the component actions and the sensory motor capabilities required to progress through the activity and achieve the end goal [17]. Thus, the activity is described in terms of a series of steps in which each step is decomposed into further sub-steps, an approach sometimes referred to as hierarchical task analysis (HTA) [18]. Typically, HTA yields a number of choice points at which different alternative sequences of action steps are possible. For example, preparing a hot drink, such as a cup of tea, can be successfully completed in a number of ways. Thus, the point in the sequence at which to add ingredients such as milk or sugar (e.g. before or after the hot water) is a personal choice. In effect, several task models are needed to accommodate the variety of ways a particular ADL task might be successfully completed. Alternatively, the task model may be represented in terms of a Markov Decision Process (MDP). In this, the series of steps in the task are considered as states and the model includes a specification of probabilities of transitions between states that allow successful completion of the task [19]. Errors may then be identified by violations of these steps. In ADL task performance, two important classes of errors should be distinguished- according to whether or not the error prevents completion of the task [20]. Recoverable errors, such as forgetting to add milk or sugar, can be corrected, for example by giving an alerting cue. Non-recoverable errors, such as adding milk when it is not required, cannot be corrected.

Once a task model has been defined, the therapist can give feedback on the succession of action steps, as they occur during ADL performance, by comparison with those specified in the task model. If carried out in real time, such corrective guidance can assist with learning the sequence of steps required to complete the task. The feedback can be delivered on a trial and error basis, only offering feedback when an error has been committed. Alternatively, an errorless learning approach may be favoured in which prospective cueing of the next step is provided with the aim of preventing errors from occurring. It has been suggested that patients with severe cognitive impairment may benefit more from the errorless learning approach than from error-based learning [21, 22].

The study reported in the present paper examines the ADL task of preparing a cup of tea. The aim was to determine the efficacy of rehabilitation of stroke patients with AADS based on a defined task model combined with a specific regime of trial-and-error training with auditory and visual feedback prompts. Making a cup of tea was chosen in the present study because it was a task that focus groups identified as important to stroke survivors [23], lay within the potential capability of the patient sample (stroke survivors with mild cognitive impairments) and, at the same time, has a structure appropriate for a task model approach to training. Thus, tea making can be broken down into various sub-actions required to complete the task [18, 24] and performance is quantifiable in terms of recoverable and non-recoverable errors. The task model was represented as an MDP with a set of states and transition probabilities (allowing alternative paths through the sequence of steps) that were obtained from observations of control participants [19]. The task model was implemented as a subset of a computer-based system [25] developed in a project, CogWatch (https://more.bham.ac.uk/cogwatch/) to provide home-based rehabilitation of ADL tasks. When this subsystem was previously tested with twelve stroke survivors with AADS, the success rate in making a cup of tea improved to 96% compared to 68% without the subsystem [25]. The present study seeks to extend this finding with training over a number of sessions, in a randomized controlled design, and including follow-up assessment.

A randomized controlled crossover design was employed. Participants meeting the entry criteria were randomly assigned to one of two groups. In one group participants first experienced tea making training and then the control condition. In the other group participants were first exposed to the control condition followed by tea making training. The control condition involved stepping in place in time to an auditory metronome providing rhythmic auditory stimulation (RAS) [26]. RAS has been shown to be an effective rehabilitation procedure for hemiparetic gait after stroke [27, 28]. This task was chosen as a placebo form of control. Thus, the task focused on the lower limb in a manner relevant to most of the participants’ needs, brought participants into the same environment as in the experimental condition (hence controlling for any associated effects), but, in itself, was considered unlikely to affect hemiparesis of the upper limb and hence, performance of the tea making ADL task. In a complementary manner, the tea making training (unlikely to affect stepping) may also be considered as a placebo control for stepping in place training.

The research question addressed was: Following tea making training do stroke survivors with AADS reduce the number of errors in tea making? Errors were described in terms of neuropsychological categories, such as step omission, step addition, sequence error, commonly found in the apraxia literature [3], rather than in terms specific to tea making. In this way, it is hoped that the findings may be compared with other ADL tasks. To check whether improved accuracy in tea making was only achieved at a cost of slower performance, assessment included time taken in tea making. Improvement in performance on both measures (reduced errors and reduced time) would then indicate that the task model approach to training tea making was effective for AADS. A complex tea making task involving preparing two cups of tea at once was also included to test for generalizability to a more complicated untrained task.

If a significant improvement in tea making performance were to be found in the present study, it would be important to know whether the improvement relates to other aspects of motor recovery, such as improving upper limb function, occurring after stroke. The analysis of the results therefore included an Explanatory variable analysis. In this, changes in motor recovery (assessed by Fugl Meyer Upper Limb and grip strength measures before and after training) were compared in patients who exhibited improvement vs no improvement as a result of the training. In addition, it was also considered interesting to determine whether improvement related to initial status of the participants, for instance, years post stroke. Tests were therefore also carried out for possible differences in baseline characteristics (age, sex, handedness, years post stroke, Nottingham Extended ADL Scale, NEADL) between the patients exhibiting improvement and no improvement.

## Methods

A within-subjects crossover design was employed; participants were randomly assigned to one of two groups in order of recruitment. Prior to the start of the trial, an Excel random number generator was used to allocate participant identification numbers 1 to 30 with equal numbers to Group 1 or 2 and the allocation was placed in 30 sealed envelopes by ES. On recruitment, each participant received a participant recruitment number which BD used to select the envelope containing their Group number. Given the design of the study, it was possible to apply a single-blind strategy where the assessors were blinded to the intervention whereas the patient was aware.

**Fig 1:**
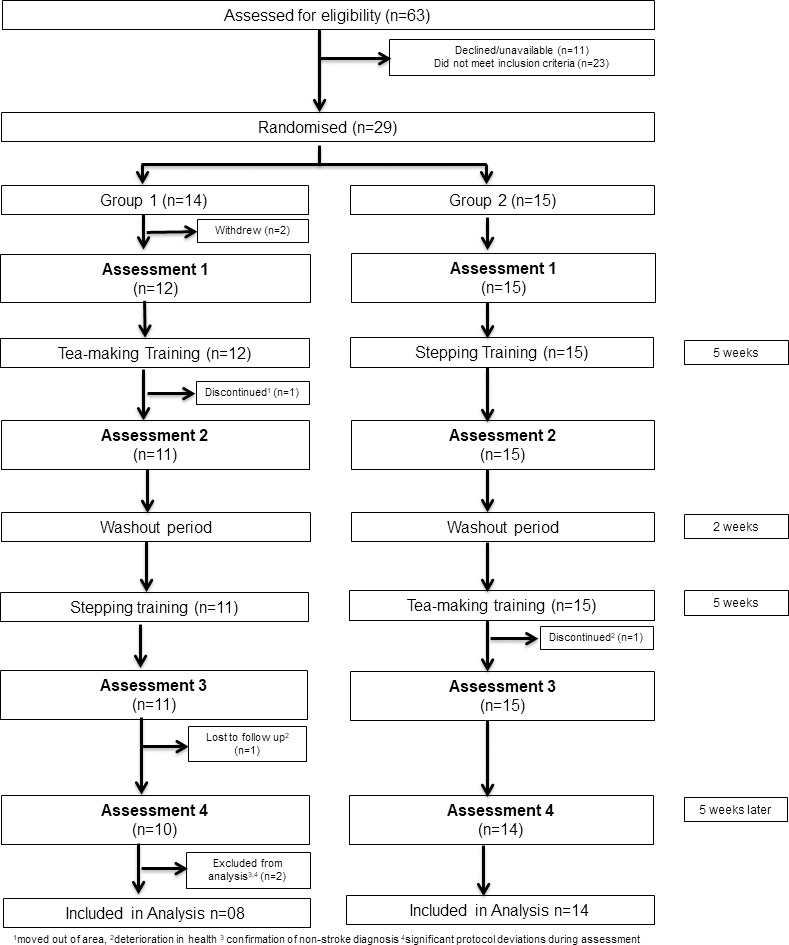
Consort diagram showing the flow of participants through the study.

Group 1 commenced Phase 1 with tea making training and Group 2 commenced Phase 1 with the stepping training control condition. Following a 2-week “wash-out” period at the end of Phase 1, in Phase 2 both groups received the alternative training, so Group 1 received stepping training and Group 2 received tea making training. A pre-post assessment design was used with 4 assessment sessions: 1) baseline (week1); 2) following Phase 1 training (week 7), 3) following Phase 2 training (week 15) and 4) follow-up (week 21). The flow of participants within the trial is summarised in Fig 1. All training and assessment sessions took place at the University of Birmingham. Ethical approval was granted by the University of Birmingham ethics committee under ERN_12-0683B (28 May 2014) with protocol retrospectively registered as a clinical trial (https://clinicaltrials.gov/ct2/show/NCT04044911?term=Cogwatch&rank=1).

### Participants

63 community dwelling stroke survivors, who had been recruited as part of the CogWatch project, were assessed for eligibility for the trial. All the stroke survivors had visited the University of Birmingham previously to participate in a range of activities including functional brain imaging and psychometric evaluation (including making a cup of tea in some cases). None had previously been trained on tea making. Of the 40 people meeting screening criteria (≥ 18 years of age; ≥ 2 months post incident; medically stable; and failing at least one of four praxis items in the Birmingham Cognitive Screen (BCoS) [29] or a document filing task which had previously demonstrated impaired task performance for stroke survivors with praxis problems [30], 29 stroke survivors gave written informed consent to participate in the study which took place between 1 June 2014 and 4 December 2014. A target of 30 participants, 15 in each Group, was determined on the basis of a power analysis (g-power) assuming a small to moderate effect size of 0.3 for detecting change in tea-making errors, as being clinically meaningful, alpha of 0.05, and a power of 0.85, allowing for approximately 25% drop-out. Screening tests also included an activity of daily living scale (NEADL) [31], and a gesture production task used to corroborate handedness (pantomime task) [32]. Baseline participant characteristics are summarized in **Table 1**.

**Table 1:** Baseline participant characteristics including patients whose data were imputed (23–25).

No	Grp	Age	Sex	Lesion^a^	Hand pref pre stroke^b^	Hand pref post stroke^c^	Years post stroke	NEADL	FM	ANX	DEP
**1**	1	80	M	unconf	right	right	>1	28	9	7	11
**2**	1	75	F	unconf	right	right	>2	49	5	9	4
**3**	1	67	F	unconf	right	left	>5	42	20	5	12
**4**	1	53	F	left*	right	right	>2	45	10	1	3
**5**	1	72	M	right*	right	right	>5	42	20	4	2
**6**	1	66	F	right*	right	right	>10	33	20	11	9
**7**	1	56	M	left*	right	left	>1	30	14	4	9
**8**	1	67	M	right*	left	right	>10	49	3	10	10
**9**	2	67	M	left*	left	left	>5	35	16	14	12
**10**	2	79	M	right*	right	left	>2	10	4	6	9
**11**	2	68	M	right*	right	left	>6	28	1	4	5
**12**	2	75	F	bilat*	right	right	>1	38	1	2	1
**13**	2	67	F	right*	right	right	>2	32	NA	4	0
**14**	2	77	M	unconf	right	left	>1	16	15	4	1
**15**	2	64	F	left*	left	left	>5	48	2	6	11
**16**	2	69	F	right*	right	right	>5	49	18	2	5
**17**	2	77	F	right*	right	right	>2	51	12	10	5
**18**	2	65	F	bilat*	right	right	>10	45	13	1	7
**19**	2	78	M	left*	left	left	>10	24	15	3	2
**20**	2	82	M	unconf	right	right	>5	54	20	4	4
**21**	2	68	F	unconf	left	both	>10	40	13	7	4
**22**	2	79	M	left*	right	right	>2	47	14	6	6
**23**	2	64	M	unconf	n/a	n/a	4	35	5	4	6
**24**	1	81	M	unconf	n/a	n/a	8	41	9	7	6
**25**	1	81	M	unconf	n/a	n/a	1	40	20	0	2

^a^* lesion site confirmed by MRI

^b^ Handedness through self report

^**c**^ Handedness from gesture production task

d NEADL max (normal) score 66

e FM max (normal) score 18

f ANX max score 21, below 8 normal

g DEP max score 21, below 8 normal.

### Assessment

Four assessment sessions covered the performance of (A) simple (1) and complex (2) tea making, (B) motor capacity; Fugl-Meyer short form [33] (3), (C) psychological state; Hospital anxiety and depression scale (HADS) [34] anxiety and depression (4), (D) stepping in place and other measures of balance and gait (5–9). Measures of performance of tasks in categories A-D are summarised in the (**S1A Table**).

**Fig 2:**
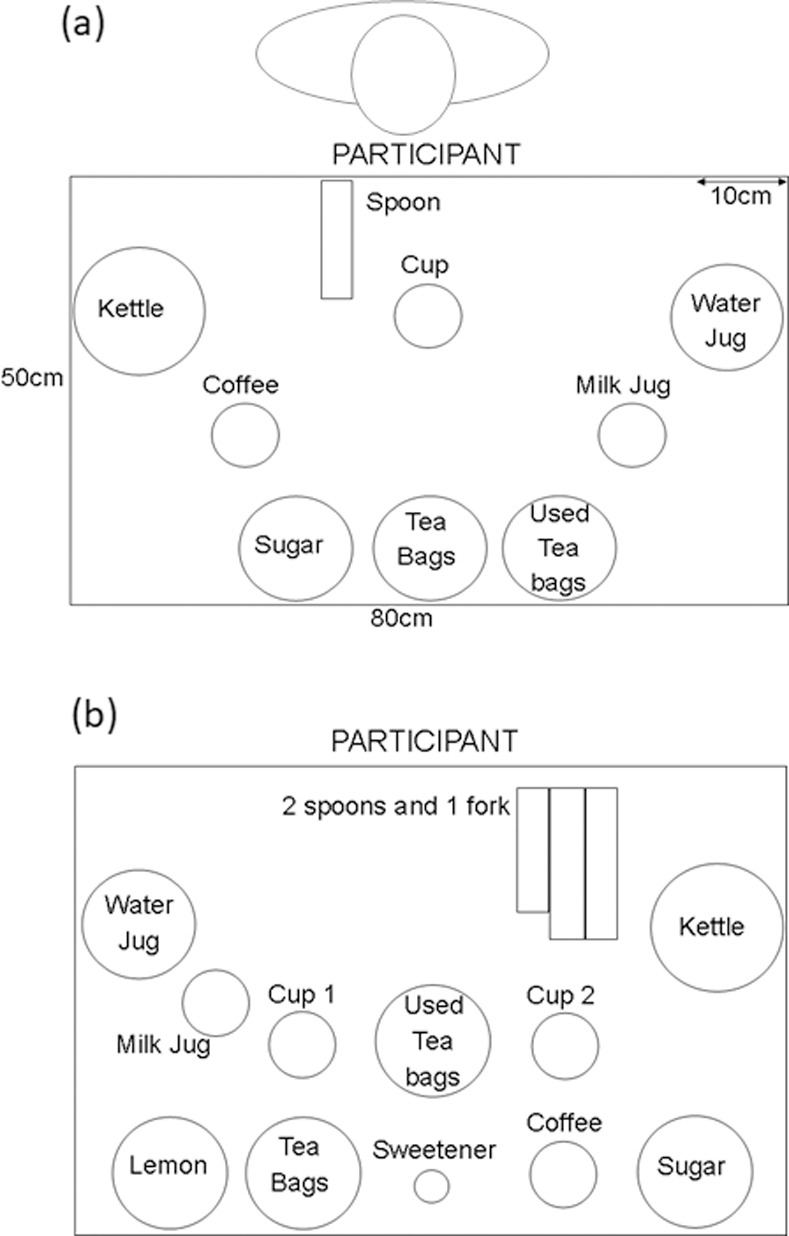
Object layout for simple tea at assessment. (a) Simple and (b) Complex Tea Layout Mat to scale (80x50cm); kettle (15cm diameter) bowls & water jug (12.8cm), cups, milk and coffee (8cm), cutlery (13.7–17.7cm), sweetener (4.5cm). Coffee was used as a distractor.

For the Simple and Complex Tea tasks participants were seated at a table with the required objects and ingredients placed in standard positions (Fig 2). A coffee jar was included as a distractor to simulate a real-life setting with multiple objects in the kitchen. Different layouts for the two tasks were used to reduce possible transfer effects. Each trial was recorded with a video camera (Toshiba Camileo 100, Tokyo, Japan) to allow subsequent classification of error performance.

For Simple Tea, which was run first, participants were instructed to make 8 cups of tea, one at a time, comprising two of each of the following; black tea, black tea with sugar, tea with milk and tea with milk and sugar (BT, BTS, WT, WTS). Participants were permitted to use both hands and ask for assistance with stabilising objects if necessary. The order of the 8 cups was randomized for each participant. No feedback was given during the tea tasks, but self-correction of errors was permitted. No time limit was set for the task, though time taken was recorded. A break was given after trials 4 and 8 of Simple Tea.

For Complex Tea, participants were provided with verbal and visual instructions to make two cups of tea simultaneously per trial (for a total of 2 trials); one cup with tea, lemon and 1 sugar cube and the other with tea, milk and 2 sweeteners. Other than changes in tea type, the instructions provided were identical to Simple Tea.

### Tea making training

**Fig 3:**
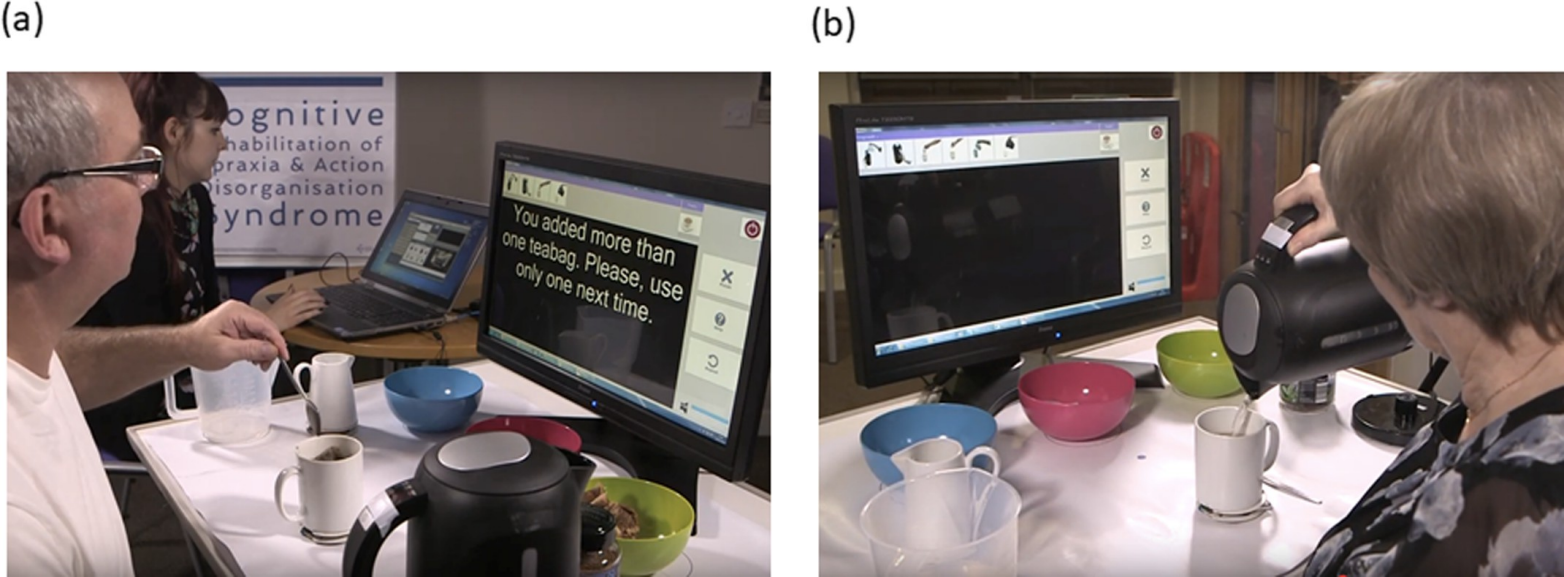
CogWatch system setup. a) As the patient completes tea-making with the items on the table layout, cues are presented on the “patient display” (foreground) when an error is detected (in this case non-recoverable). The healthcare professional monitors task progression via the “clinician display” (background). b) Each correct step is acknowledged by the system by displaying a visual reminder of the completed sub-action at the top of the patient display. Here, the patient has completed “Add water from jug to kettle”, “Boil water”, “Add teabag to cup”, “Add sugar”, “Add milk” and is performing the sub-action of “Add boiled water to cup”. We acknowledge The Stroke Association, UK, as the source of this figure. The individuals in this figure have given written informed consent (as outlined in the PLOS consent form) to publish their image.

Training of tea making used the apparatus and table layout as described under the Simple Tea assessment together with the addition of 2 displays required by the CogWatch subsystem (Fig 3). One of these displays, the ‘clinician display’ (not visible to the participant), allowed the experimenter to manually identify actions using video feed from a Kinect ™ sensor (Microsoft, Redmond, USA). The possible sub-actions comprised: add water from jug to kettle, boil water, add teabag to cup, add boiled water to cup, add sugar to cup, add milk to cup, stir, remove teabag. The other display, the ‘patient display’, provided the participant with task progress information, error feedback (when required) and an option to obtain assistance.

The CogWatch subsystem software comprised state modeller, action policy module, error recognition module, and cue selector. Experimenter manual data entry was used to record completed action steps. The subsystem provided feedback to the participant according to whether each step was correct or in error (by reference to a look-up table of errors, i.e. violations of action sequences permitted by the MDP task model). If the error was recoverable (e.g. failure to add milk), the subsystem cued the participant for this action at the end of the task. If the error was non-recoverable (e.g. sugar added when not required), the task was terminated at that point. If the participant failed to take any action within 30 s of the previous step, the subsystem cued the most probable next step based on state transition probabilities estimated for data previously collected from healthy normal control participants. There was also an option for the participant to request cueing of the most probable next step. Cues comprised a brief video clip of the required action (e.g. a sugar cube is taken and placed in the cup) together with a simple audio request (e.g. “please add sugar”).

Participants attended five 1-hour weekly sessions in which the CogWatch subsystem was used to train their tea making. For each session, participants were required to make 8 cups of tea, as in the Simple Tea Task (2 each of BT, BTS, WT, WTS). The order of tea types was randomized for each session for each participant. A compulsory break was given after 4 cups, with the option of additional breaks where required.

Participants were instructed to make a cup of tea as they normally would at home, but to respond to cues appearing on the ‘patient display’. The CogWatch subsystem was used in error-based training mode in which the system displayed a multimodal cue (video with superimposed text and spoken instructions) for the required action for any of the following 3 reasons: (1) the participant pressed the help/repeat button (using the interactive touch screen buttons), (2) more than 30 s elapsed since the previous action (except when the kettle was boiling, in which case approximately 30s after the kettle had finished boiling, (3) the participant made an error (i.e. task model violation). In each case, the cue would instruct the participant on what to do next (i.e. the most probable sub-action based on normative data). If a non-recoverable error was made, the subsystem informed the participant of the error type (e.g. ‘you added sugar when it was not required’), told them to take a break and ended the trial. If 3 successive cues were displayed, the trial would also be terminated.

**Fig 4:**
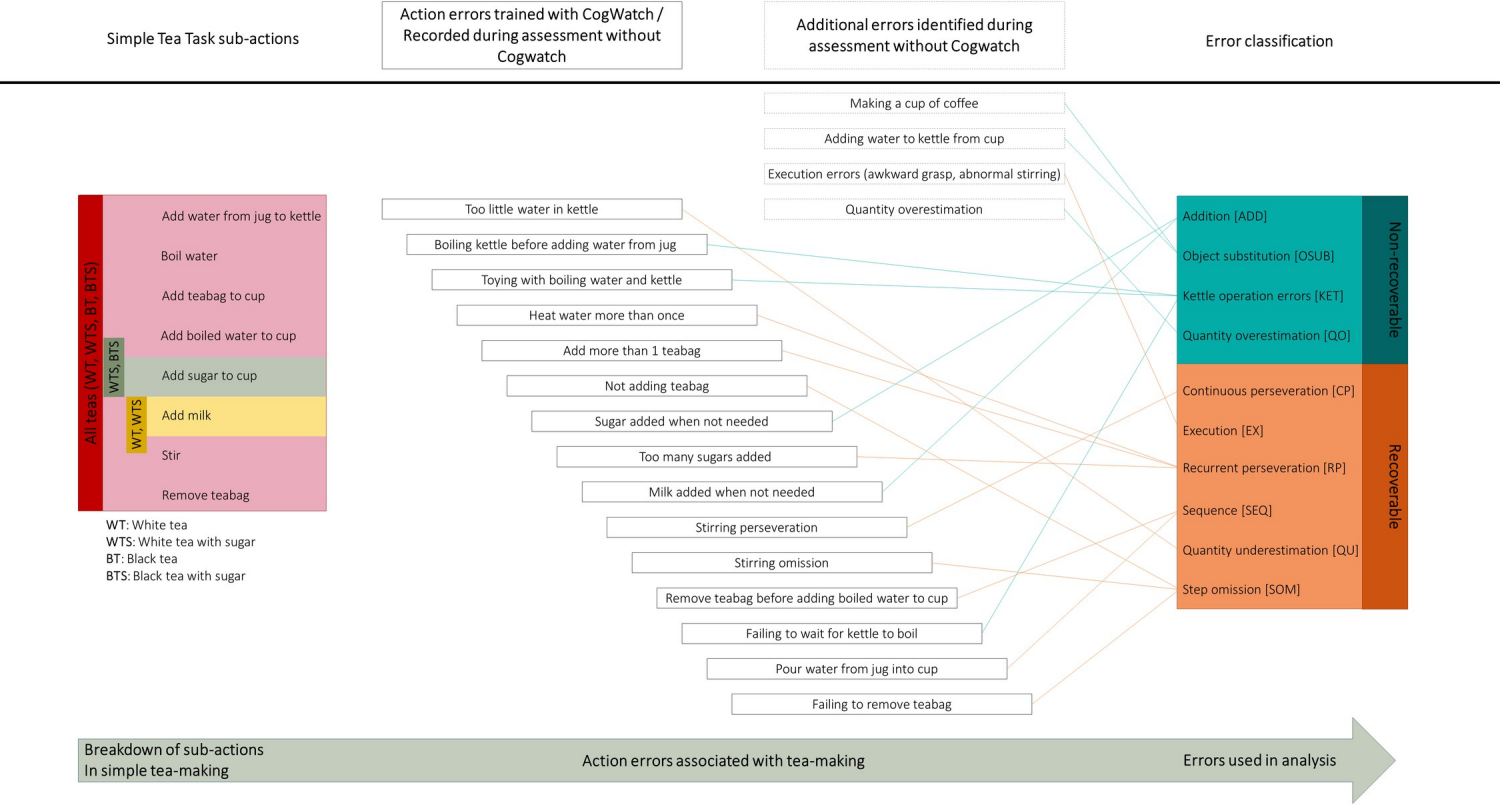
Mapping of actions and errors to neuropsychological classification. From left to right are the sub-actions which are performed during simple tea making, action errors which may result during performance of the sub-actions, and their relation to the neuropsychological error categories which were used for analysis. In addition to errors which were trained by the Cogwatch subsystem, 4 additional errors were identified during the assessment (in boxes with broken line borders).

The CogWatch subsystem required the experimenter to identify the sub-actions performed by the stroke survivor in real time, using a set of ‘action buttons’ on the ‘clinician display’. For example, when water was added from the jug to the kettle the experimenter confirmed successful completion of this sub action by pressing the appropriate ‘action button’. A cue was displayed to the participant if the confirmed action was not part of the predefined action sequences in the CogWatch tea making task model. For example, if ‘boil water’ was identified before ‘add water to kettle’, the system recognised that the participant had violated the task model and produced a cue to alert the participant to their mistake. Action errors detected during training are summarised in Fig 4.

### Stepping training

Participants were instructed to step in place to an auditory cue in each of five 1-hour weekly sessions. Participants who were wheelchair users remained seated with heel resting on the ground and made ankle movements to lift and replace the forefoot in place of stepping. A tablet computer (iPad mini™ Apple, Cupertino USA) with preloaded popular music in digital MIDI format was used to define a stable regular pulse comprising a metronome beat overlaid with music. The tracks were digitally varied to participants’ abilities, providing tempo and phase shift changes at a range of speeds from 45–105 beats per minute, so that the training encouraged adaptability of lower limb movement [28].Training was administered in three blocks of 10 minutes, interspersed with rest periods. Over participants, tempo ranged between 45–105 beats per minute.

### Data analysis

The frequency and type of errors made during the simple and complex tea tasks were recorded in a bespoke Microsoft Excel datasheet. **Table 2** provides a description of error types used in the analysis together with illustrative examples. These neuropsychological errors relate to the errors defined within the CogWatch subsystem as shown to the right of Fig 4.

**Table 2:** Error types and definitions.

	***Non-recoverable Errors***		
	**Error type**	**Definition**	**Example**
1	Addition	Adding an extra component action that is not required in the action sequence.	Adding sugar or milk when not required.
2	Object substitution	An intended action carried out with an incorrect object.	Adding water to kettle from cup instead of water jug; adding coffee instead of tea.
3	Kettle operation error	Kettle not operated correctly.	Failing to add water to kettle; failing to turn on the kettle during task and using water; failing to wait for the kettle to boil.
4	Quantity—overestimation	Adding too much of an ingredient	Too much water added to cup causing tea to spill when stirred.
	***Recoverable Errors***		
	**Error type**	**Definition**	**Example**
1	Continuous perseveration	Inappropriate prolongation or repetition of a behaviour without interruption.	Continuous stirring of tea more than 10 s).
2	Execution	An error in the execution of an action, resulting in clumsy movement.	Awkward grasp; abnormal stirring (e.g. causing spilling of tea); stirring action outside cup.
3	Recurrent perseveration	When a step or a sequence of steps is repeated (after achieving its goal) later on in the action sequence.	Adding milk several times (interleaved with another action); repeated stirring; repeatedly adding sugar.
4	Sequence	Performing an action in the wrong order (compared to controls, or functional logic).	Removing teabag before adding water; turning the kettle on before pouring water into the kettle (self-corrected).
5	Quantity—underestimated	Adding too little of an ingredient.	Not adding enough water to the cup (under 50% full).
6	Step omission	Omitting a step.	Failing to add sugar, milk or teabag; failing to add water from kettle.

The assessment videos of the participants making simple tea and complex tea were analysed by two of the authors (JH, ES) who were blind to the session depicted in each video. The assessment sessions were assigned at random with unblinding undertaken by another member of the research team (PW) only after all videos were assessed. The average completion time and summed errors across the 8 tea making trials at each assessment were used to provide measures of speed and accuracy for each participant.

Approximately 15% of the videos (112 tea making trials) were analysed by both assessors to allow for an analysis of inter-rater reliability. There was good agreement between the two assessors as revealed by significant correlations between the raters for Total Time (Pearson’s *r* = .939, n = 112, *p* < .001); and Total Errors (*r* = .823, n = 112, *p* < .001).

Task completion time (in seconds) for each trial was determined from the video recording relative to the verbal start instruction given to the participant. Completion was defined when the participant indicated task completion verbally or when all other tea making activities ceased. The time taken for the kettle to boil varied across trials and was deducted from the completion time.

The main analysis consisting of repeated measures ANOVA was performed for group comparisons. 2 x 3 repeated measures ANOVA was conducted with error type (recoverable errors, non-recoverable errors) and contrast (experimental, control, follow-up) as factors. Experimental (before vs after tea making training), control (before vs after stepping training), and follow-up (post tea making training vs follow up assessment) contrasts are defined as differences between assessments 1–2, 2–3, and 2–4 in Group 1, and assessments 2–3, 1–2, and 3–4 in Group 2 respectively. A one-way repeated measures ANOVA was used to identify mean change in time to complete tea making between assessment contrasts (experimental, control, follow-up). Where Mauchly’s test for sphericity was significant, we report the Greenhouse-Geisser correction as the Hyunh-Feldt and lower bound corrections yielded the same results. Where ANOVA factors were significant, post-hoc pairwise comparisons were performed to identify comparisons driving the differences. For all tests, a *P* value of < .05 was considered statistically significant. We also performed an exploratory analysis to understand the contribution of explanatory variables in patients who showed improved performance after CogWatch training. All analyses were completed using SPSS v. 24 (IBM Corporation, Armonk, NY, USA).

## Results

Of the 29 community dwelling stroke survivors recruited to participate in the study, 24 completed both training interventions and all assessments. Of the five participants who did not complete the trial, two withdrew after randomisation and prior to Assessment 1. As no data were collected from these patients, they had to be excluded from the analysis. Three participants dropped out during the trial (2 from Group 1; 1 from Group 2) due to reasons unrelated to the study (broken leg, cancer diagnosis, moved away). For assessment data points which were missing, we performed the main analyses including these three patients by imputing missing data using chained equations considering available baseline data as auxiliary variables. Data from two further participants were excluded from the analysis after the trial had completed (Fig 1). One was due to the subsequent confirmation of a non-stroke diagnosis and the other was due to significant deviations in the protocol during the assessment sessions. As the tea-making assessments were video recorded for analysis, deviations in the prescribed protocol were highlighted by the blinded assessors during the analysis phase of the trial. Therefore, a total of 25 participants were included for main analysis. The characteristics for these participants at baseline are shown in **Table 1**. The average age was 71.1 (±SD, 7.8) years and number of years post stroke was 4.6 (±SD, 3.3) years with no differences between groups. As a sensitivity analysis, we present the main analysis without imputed data as **(S1 Text)** using only complete cases from 22 participants.

### Simple tea making

**Fig 5:**
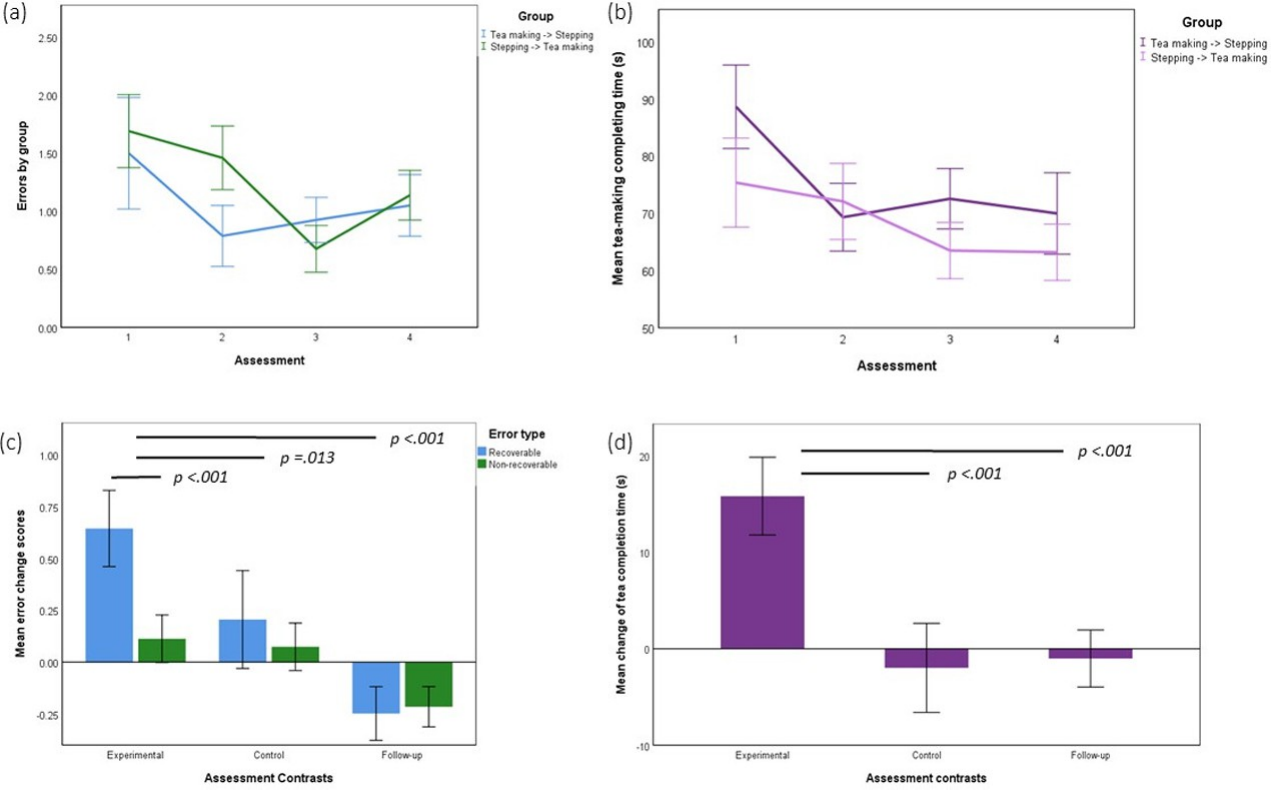
Tea making performance. (a) error (b) time taken for the two patient groups (Group 1: Baseline (1), Post-training (2), Post-control (3), Follow-up (4); Group 2: Baseline (1), Post-control (2), Post-training (3), Follow-up (4)). Tea making change scores (c) Recoverable and nonrecoverable errors (d) time as a function of condition. Experimental, control and follow-up scores were calculated 1–2, 2–3, 2–4 (where experimental, control and follow-up were calculated as Group1) and 2–3,1–2, 3–4 (Group 2). Statistically significant contrasts are shown across the top of (c, d).

Inspection of the average number of errors (Fig 5A) and time taken (Fig 5B) across the 4 assessment sessions reveals reductions due to training that exceed the reductions in the control condition, in both Groups 1 and 2.

Fig 5C and 5D show the error (recoverable and non-recoverable) change scores and time change scores respectively, for experimental, control and follow-up contrasts as previously defined.

The 2 x 3 repeated measures ANOVA identified a significant main effect of error type *F(*1,99) = 12.970, *p* < .001 and assessment condition *F*(1,198) = 18.926, *p* < .001. There was also a significant interaction between error type and contrast *F*(1.855, 183.692) = 6.788, *p* = .002 (Greenhouse-Geisser correction, Ɛ = 0.918).

Post-hoc pairwise comparisons indicated that there was a larger overall number of recoverable errors (mean±SE .201±.058) compared to non-recoverable (-.010±.033; *p* < .001). The total number of errors reduced between experimental (.379±.057) and control contrasts (.140±.066; *p* = .013), between experimental and follow-up contrasts (-.237±.040; *p* < .001), as well as between control and follow-up contrasts (*p* < .001). The interaction effect was driven by differences between recoverable (.645±.092) and non-recoverable errors (.113±.057; *p* < .001) in the experimental contrast but not in the control (*p* = .318) and follow-up (*p* = .687) contrasts.

The one-way repeated measures ANOVA with Greenhouse-Geisser correction (Χ^2^(2) = 41.010, *p* < .001) indicated that mean change in time to complete tea making was significantly different between assessment contrasts (experimental, control, follow-up) *F*(1.490, 147.547) = 21.094, *p* < .001. Post-hoc pairwise comparisons revealed that time reduced between experimental (mean±SE 15.826±2.015 s) and control (-1.983±2.309 s; *p <* .001) as well as experimental and follow-up (-1.014±1.481 s; *p <* .001) contrasts but there was no significant difference between control and follow-up contrasts (*p* = .724).

**Fig 6:**
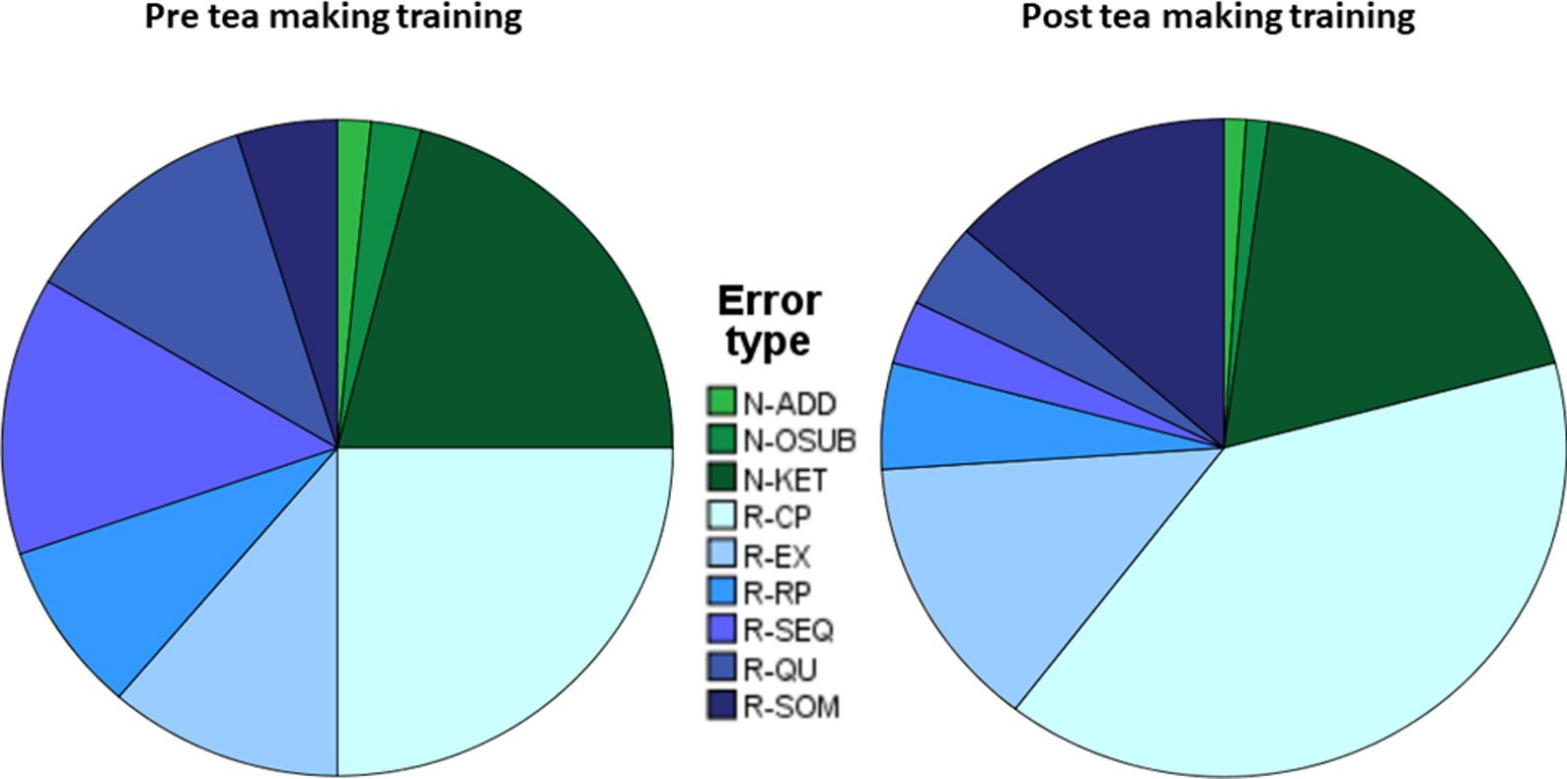
Error changes. Number of three types of non-recoverable (addition N-ADD, object substitution N-OSUB, kettle error N-KET) and six types of recoverable errors (addition N-ADD, object substitution N-OSUB, kettle error N-KET) before (dark shade bars) and after tea-making training (light shade bars). Note that only data from complete cases (n = 22) are included in this figure as error type data were not imputed.

We now provide a descriptive analysis of error types where only complete case data from 22 patients were used. Fig 6 shows the changes of non-recoverable and recoverable errors before and after tea making training. Prior to training, the most frequently observed errors were for N-KET (kettle errors) and R-CP (continuous perseveration). Post-training, the frequency of errors decreased for all error types except R-SOM (step omission).

**Fig 7:**
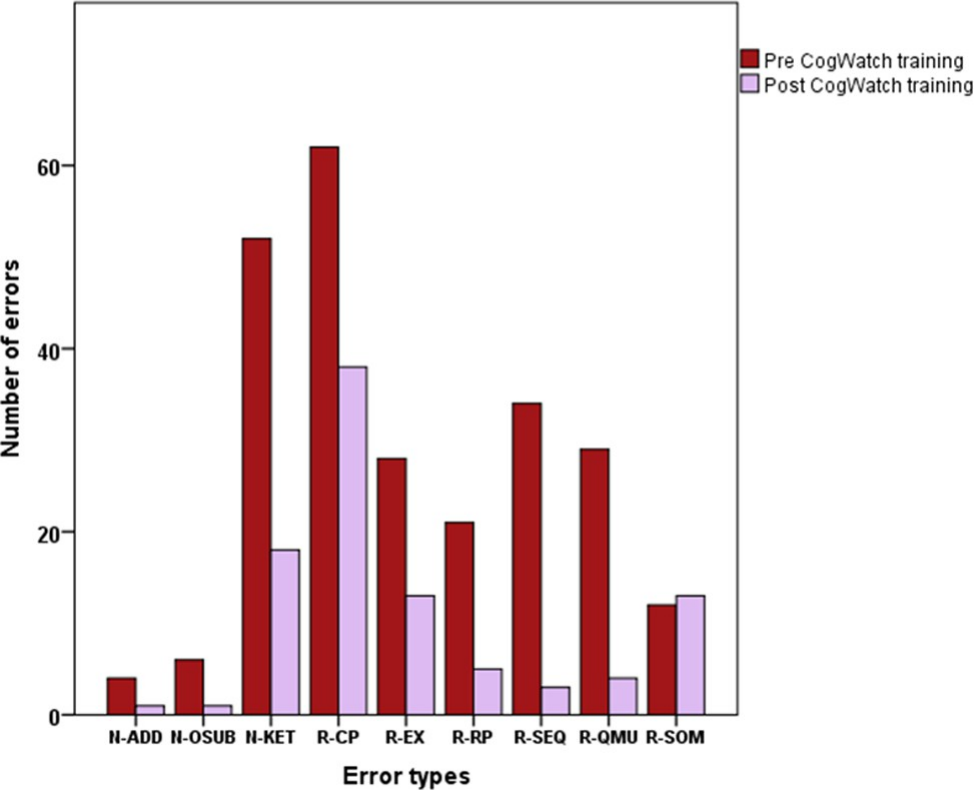
Error types. Relative proportions of the three types of non-recoverable errors (addition N-ADD, object substitution N-OSUB, kettle error N-KET in shades of green) and six types of recoverable errors (continuous perseveration R-CP, execution R-EX, recurrent perseveration R-RP, sequence R-SEQ, quantity underestimation R-QU, step omission R-SOM in shades of blue) pre and post-tea making training. Note that no non-recoverable step omission errors occurred during the pre and post-tea making sessions.

We visualise the same errors in a pie chart (Fig 7) which emphasizes that the relative proportions of non-recoverable error types (N) remained relatively constant, (when the number of non-recoverable errors decreased relatively little, Fig 5). However, in the case of recoverable errors (R), (where Fig 5 shows a relatively large decrease), the reductions in errors resulted in relatively greater proportions of R-CP (continuous perseveration), R-QMU (quantity underestimation) and R-SOM (step omission).

### Complex tea making

**Fig 8:**
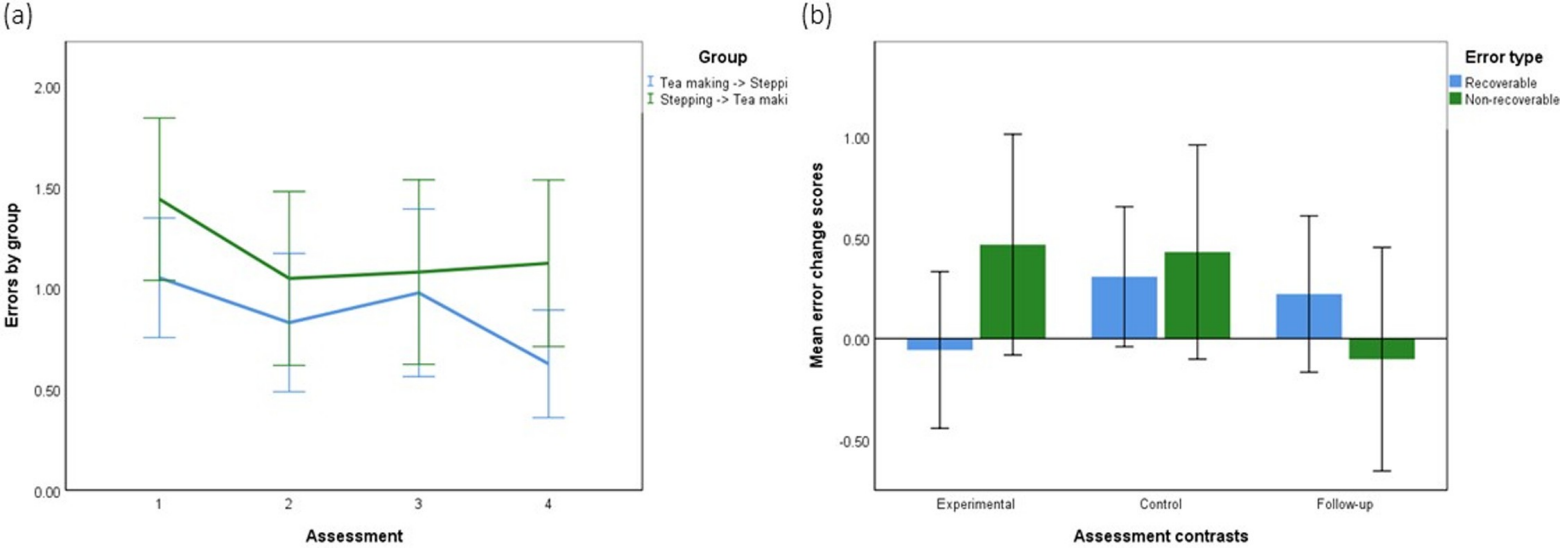
Complex tea making. (a) Proportion of complex tea making errors as a function of training condition. (b) Mean change in complex tea making errors across contrasts.

Fig 8A shows the number of errors in the untrained complex tea making task as a function of error type (recoverable vs non-recoverable) for the two groups (c.f. Fig 5A). This shows a reduction of errors in group 1 before and after simple tea making training (session 2 vs 1). However, group 2 also shows a reduction before and after the control condition and no reduction from session 2 to 3 associated with simple tea making training.

Fig 8B shows the error (recoverable and non-recoverable) scores as a function of experimental (before vs after tea making training), control (before vs after stepping training) and follow-up (post tea making training vs follow up assessment) contrasts (Group1 assessments 1–2, 2–3, 2–4 and Group 2 assessments 2–3, 1–2, 3–4) respectively (c.f. Fig 2A). 2 x 3 repeated measures ANOVA was conducted with error type (recoverable errors, non-recoverable errors) and contrast (experimental, control, follow-up) as factors. There was no significant main effect of error type (*p* = .549) or assessment condition (*p* = .405; Greenhouse-Geisser correction).

### Exploratory analysis

#### Baseline characteristics

Using complete cases only, baseline characteristics between patients who improved and did not improve post tea making training were compared. Of the characteristics analysed (age, sex, handedness, years post stroke, NEADL) only age was significantly associated with increased odds of improvement outcome (OR = 1.250, 95%CI = 1.002, 1.559, see **S1B Table**).

#### Explanatory variables

To understand if motor recovery or grip strength contributed to improvements in tea making performance, we analysed the Fugl-Meyer upper limb scores and grip strength performance to determine if they were independent of the effects of tea making training.

At baseline, neither the Fugl-Meyer upper limb score nor grip strength were associated with improvements post-training (Fugl-Meyer Odds Ratio, OR = 0.907, 95% confidence interval, CI = 0.774, 1.107; grip strength OR = 1.025, 95%CI = 0.887, 1.186).

There were also no significant differences between the Fugl-Meyer upper limb scores pre-and post-training (*p* = .473; median [IQR] pre-training 11 [0.25–12.00]; post-training 11 [2.25–12.00]. A similar outcome was observed for grip strength performance pre-and post-training (*p* = .267; mean [SD] pre-training 23.37 [6.55]; post-training 24.79 [7.16]).

## Discussion

Cognitive disorders of movement after stroke, in particular Apraxia and Action disorganization syndrome (AADS), can impair activities of daily living (ADLs) such as preparing a hot drink. Occupational therapy (OT) for stroke typically comprises: assessment, goal setting, training of ADLs, transfers and mobility(10). The aim of the present study was to test the efficacy of a task model approach to retraining tea making in AADS. In a cross-over randomized design, the effects of tea making training on accuracy and speed of tea making was contrasted with the effects of a control condition involving stepping training. The tea making training was based on monitoring progress and giving feedback on the accuracy of subtask completion.

Improvements in time taken and accuracy of tea making across black or white (with milk) tea with or without sugar were reliably greater following the experimental tea making condition compared to the stepping control condition. These improvements in performance were well sustained at the end of the study (between 7 and 15 weeks post tea making training), with errors and time taken at follow-up reduced by 25% and 18% of pre-training values. In many tasks, improvements in speed are achieved at the cost of a reduction in accuracy [35]; however, this was not the case here as both errors and speed reduced with training. The improvement observed in simple tea making in the present study suggests the use of the task-based approach in future research to explore the effects of different training parameters on the efficacy of ADL rehabilitation.

Errors were classified as recoverable or non-recoverable. There were more recoverable errors than non-recoverable errors. Recoverable errors decreased significantly after training compared to the control condition, whereas there was no significant decrease in non-recoverable errors. Recoverable errors comprised: continuous perseveration, execution, recurrent perseveration, sequence, quantity underestimation, step omission. Non-recoverable errors included addition, object substitution, kettle operation. Prior to tea making training the most common type of recoverable error was continuous perseveration, and kettle operation was the most frequent type of non-recoverable error. The relative proportions of sub types of non-recoverable errors were similar before and after training. However, after training, there were relatively greater proportions of continuous perseveration, quantity underestimation and step omission (Fig 7). Focusing training on these three error types could therefore be an appropriate focus for future research into ADL training interventions.

The study protocol included complex tea making (simultaneously making two different cups of tea) as part of the assessment. The results did not indicate transfer from simple tea making training to complex tea making. This contrasts with Geusgens et al. [36] who reported transfer of training on one of three ADL tasks (washing, dressing, preparing a sandwich) to a fourth untrained task (making a cup of hot chocolate). Interestingly, these authors also reported that strategy training (in addition to usual therapy) increased the transfer effect. The authors represent the strategy as using internal (self-verbalisation) or external (using pictures to illustrate the required action sequence) compensation for the impairment. However, a previous study has shown that, if strategy training is to be effective, it needs to be in combination with performance of ADL tasks. Thus, Goldenberg et al. [20] found no benefit in training ADL activities, such as preparing coffee, after teaching structure-function relations underlying correct performance if there was no completion of the activity. Moreover, a recent Cochrane review on interventions for apraxia [37] concluded that not enough evidence exists to support or refute their effectiveness and that there is a need to develop task specific training i.e. the training of specific ADLs.

The improvement in tea making performance was evaluated against explanatory variables relating to motor recovery (e.g. grip strength) and participant status (e.g. years post stroke) at study onset. No difference was found between participants who improved on tea making versus those who did not improve in terms of change in Fugl Meyer upper limb score or grip strength during tea making training. This suggests general motor recovery during tea making training did not underlie tea making improvement. However, a difference between the two patient groups was found in terms of age, with increasing age being associated with tea making improvement. This supports the inclusion of older participants in future tests of the task model approach to ADL retraining. To understand why this effect arose would need an examination of possible interaction with the other variables; however, the small N does not warrant the use of multivariate regression at this stage. In future research directed at validating our findings with other datasets, it will be important to include larger cohorts to allow exploration of interaction between variables.

Tea making training in the present study involved a task model approach with the steps in the action sequence represented as a Markov Decision Process. When the experimenter entered actions indicating that participants had diverged from permitted sequences of actions, this was recognised as an error by software within CogWatch, a system developed for home-based rehabilitation of ADL tasks (https://more.bham.ac.uk/cogwatch/). Audio, visual and text cues were then given to draw the participant’s attention to the error. While such trial and error learning is considered effective for some participants, for the more severely impaired participant, prospective cueing, in which the participant is informed of the next required step, may be considered preferable [21].

### Clinical implication

It is widely agreed that providing stroke survivors with more therapy improves outcomes [38, 39]. Therefore, to optimise the rehabilitation trajectory, it is advised that stroke survivors engage in intensive practice of the particular task being targeted [10, 40]. The UK national clinical guidelines for stroke recommended that stroke survivors receive a minimum of 45 minutes of rehabilitation per discipline each day, though this can be incrementally accrued throughout the day allowing for fluctuations in post stroke fatigue [10].

Some stroke services find it challenging to deliver daily rehabilitation at the recommended intensity as evidenced by the national variation in delivery of rehabilitation [41]. Service pressures, staff absences and difficulties in recruiting to certain professions can all negatively impact upon the ability of services to deliver intensive rehabilitation to stroke survivors in their care. Adopting an automated MDP approach to the rehabilitation of specific ADLs could help some stroke services increase the amount of therapy delivered, especially if rehabilitation assistants are used to oversee the process. Rehabilitation assistants are commonly employed within stroke services and under the direction of a qualified therapist deliver rehabilitation to the stroke survivor, leaving the qualified therapist to deliver rehabilitation to other patients. The use of such decentralized models of therapy have been accelerated by the Covid-19 pandemic and can be feasibly leveraged to broaden access to neurorehabilitation in the long-term [42].

In recent literature, ADLs have been classified into three areas; personal ADLs (PADLs) domestic ADLs (DADLs) and extended ADLs (EADLs). PADLs encompass a variety of personal activities such as those associated with personal hygiene, (bathing, toileting and dressing) as well as eating and drinking. DADLs typically involve activities such as those found in meal preparation or home management whereas EADLs involve activities such as shopping, work or driving. Future research should seek to generalise findings from a specific area across different types of ADLs to maximise the impact of rehabilitation.

### Limitations

The primary limitation of the present study was small N preventing multivariate analysis which would have allowed exploration of interactions between variables. In future studies with increased sample size, it will also be interesting to evaluate the efficacy of different forms of feedback cueing in tea making, for example to contrast trial and error learning with errorless learning. An additional imitation of this study was that stroke survivors included in the research were not reassessed at the end of the training phases to determine if tea making or gait training had resulted in any improvements on their AADS scores. However, this is in line with rehabilitation which focuses on the attainment of personalised functional goals. Evaluation of the modality (auditory vs visual) and form (static image vs video) of the feedback would also be important with a view to improving the effectiveness of the CogWatch system. Finally, we acknowledge the importance of masking and allocation concealment in conducting the trial [43]. We have conducted this trial with the appropriate blinding by keeping the assessors and data analysts unaware of the assigned group for all participants in adherence to the requirements of our local ethics governance and CONSORT guidelines for non-pharmacological treatments. In future, trials could also explicitly assess the success of blinding by asking blinded personnel to guess the randomisation group and comparing it to responses expected by chance [44] although it is unclear how such blinding assessment results should be interpreted [45].

## Conclusion

In summary this randomized controlled trial has demonstrated that, in stroke patients with apraxia and action disorganization syndrome (AADS), tea making training with trial and error feedback is effective compared to a stepping control condition in reducing errors and time taken to make cups of tea. The improvements endured through to follow-up 7–15 weeks post training. Further research is needed to identify the relative importance of the various components of the training to the improvement.

## Supporting information

S1 Table Supporting tables A and B. (DOCX)

S2 Table CONSORT checklist. (DOCX)

S1 Text Supporting analyses. (DOCX)

S1 File Ethical review and appendices. (PDF)

S2 File Ethical review amendment. (PDF)

## List of abbreviations

AADS: Apraxia and action disorganization syndrome
ADL: Activities of daily living
BT: Black tea
BTS: Black tea with sugar
HADS: Hospital anxiety and depression scale
HTA: Hierarchical task analysis
MDP: Markov Decision Process
NEADL: Nottingham Extended ADL Scale
OT: Occupational therapy
RAS: Rhythmic auditory stimulation
RCTs: Randomized controlled trials
WT: Tea with milk
WTS: Tea with milk and sugar
