# Supplementary material for: The efficacy of a task model approach to ADL rehabilitation in stroke apraxia and action disorganisation syndrome: A randomised controlled trial
Source: PLoS One. 2022 Mar 3;17(3):e0264678. doi: 10.1371/journal.pone.0264678 (PMC8893688; doi:10.1371/journal.pone.0264678)
Supplement: S1 Table — (DOCX) [file pone.0264678.s001.docx]

***S1_Table***

***Supporting Information***

The efficacy of a task model approach to ADL rehabilitation in stroke apraxia and action disorganisation syndrome: A randomised controlled trial.

Jo Howe^1,2^ ***^¶^***, Winnie Chua^1,3^ ***^¶^*** *, Emily Sumner^1^, Bogna Drozdowska^1^, Rosanna Laverick^1^, Rachel L Bevins^1,4^, Emilie Jean-Baptiste^5^, Martin Russell^5^, Pia Rotshtein^1^, Alan M Wing^1^.

^1^School of Psychology, College of Life and Environmental Sciences, University of Birmingham, Birmingham, UK.

^2^School of Pharmacy, Aston University, Birmingham, UK.

^3^Institute of Cardiovascular Sciences, College of Medical and Dental Sciences, University of Birmingham, Birmingham, UK.

^4^School of Life Sciences, Faculty of Health and Life Sciences, Coventry University, Coventry, UK.

^5^School of Electronic, Electrical and Systems Engineering, College of Engineering and Physical Sciences, University of Birmingham, Birmingham, UK.

*Corresponding author

Winnie Chua

Email: [w.chua.1@bham.ac.uk](mailto:w.chua.1@bham.ac.uk) (WC)

***¶*** These authors contributed equally to this work.

***Table A:*** Outcome measures taken at baseline.

| **Test Grp** | **MeasNo** | **Measure** | **Description** |
| --- | --- | --- | --- |
| A | 1 | Simple Tea Making Task; accuracy and speed. | Primary (total errors, determined by classification of video of patient making a single cup of tea) and secondary (time taken determined from the video) outcome measures. |
| A | 2 | Complex Tea Task | Error classification of video of patient making 2 cups of tea at once. Included to test the generalizability of Simple Tea making skills |
| B | 3 | Fugl-Meyer Motor Scale (Short Form)^1^ | Assessment of upper (6-items) and lower (3-items) limb movement synergies. |
| C | 4 | Hospital Anxiety and Depression Scale, (HADS)^2^. | Index of psychological state. Assessed patient anxiety and depression levels using a standardised questionnaire. |
| D | 5 | Stepping in Place* | Step in place, at a comfortable rate for 20 s used as baseline cadence for control training. |
| D | 6 | Timed Up and Go (TUG)^3^ | Assessed mobility, requiring the patient to stand up from a chair, walk 3 m along the walkway, turn and return 3 m to sit back on the chair. |
| D | 7 | 6 Metre Walk^4-6^ | Assessed gait kinematics for a 6 metre distance, using a pressure sensitive walkway (Zeno 16 x 2 feet, Protokinetics Havertown PA USA). |
| D | 8 | Fear of Falling Questionnaire^7^ | Assessed patient self-efficacy and confidence with balance, using a standardised questionnaire |
| D | 9 | Massachusetts General Hospital Functional Ambulation Categorisation^8^ | Categorised each patient according to basic motor skills necessary for functional ambulation using standardised criteria. |

***Table B:*** Descriptives table of patient characteristics and explanatory variables at baseline. Improvement is defined as improvement on time to complete tea and reduction of errors on ≥50% of tea-making trials. Univariate analysis indicated age was the only variable significantly associated with increased odds of improvement. Baseline characteristics tabulated according to primary outcome groups (patients with and without improvement). Categorical data were summarised by frequencies and percentages, n(%)^a^. Continuous data were summarised by mean and standard deviation, m(SD)^b^ if normally distributed, or median and interquartile range, median(IQR)^c^ if not normally distributed.

| **Variable** | **No improvement (n = 6)** | **Improvement (n = 16)** | ***P* value** | **Univariate analysis** | |
| --- | --- | --- | --- | --- | --- |
|  |  |  |  | **OR** | **95%CI** |
| Age, years ^b^ | 63 (5) | 73 (7) | .008 | 1.250 | 1.002, 1.559 |
| Sex, male ^a^ | 1 (16.7) | 10 (62.5) | .056 | 0.120 | 0.011, 1.288 |
| Handedness, no change post-stroke ^a^ | 5 (83.3) | 10 (62.5) | .364 | 0.333 | 0.031, 3.579 |
| Post CVA, years ^c^ | 5 (2-6) | 4 (1-9) | .641 | 0.976 | 0.737, 1.292 |
| NEADL ^b^ | 41.2 (6.3) | 36.8 (13.2) | .446 | 0.964 | 0.879, 1.057 |
| Fugl-Meyer upper limb ^b^ | 5.3 (4.4) | 6.6 (5.0) | .584 | 1.062 | 0.866, 1.301 |
| Grip strength ^b^ | 21.0 (5.8) | 23.5 (6.6) | .420 | 1.072 | 0.911, 1.261 |

***References***

1. Hsieh YW, Hsueh IP, Chou YT, Sheu CF, Hsieh CL, Kwakkel G. Development and validation of a short form of the Fugl-Meyer motor scale in patients with stroke. *Stroke.* 2007;38(11):3052-3054.

2. Zigmond AS, Snaith RP. The Hospital Anxiety and Depression Scale. *Acta Psychiat Scand.* 1983;67(6):361-370.

3. Podsiadlo D, Richardson S. The timed "Up & Go": a test of basic functional mobility for frail elderly persons. *J Am Geriatr Soc.* 1991;39(2):142-148.

4. Bohannon RW. Comfortable and maximum walking speed of adults aged 20-79 years: reference values and determinants. *Age Ageing.* 1997;26(1):15-19.

5. Bohannon RW, Andrews AW, Thomas MW. Walking speed: reference values and correlates for older adults. *J Orthop Sports Phys Ther.* 1996;24(2):86-90.

6. Wolf SL, Catlin PA, Gage K, Gurucharri K, Robertson R, Stephen K. Establishing the reliability and validity of measurements of walking time using the Emory Functional Ambulation Profile. *Phys Ther.* 1999;79(12):1122-1133.

7. Yardley L, Beyer N, Hauer K, Kempen G, Piot-Ziegler C, Todd C. Development and initial validation of the Falls Efficacy Scale-International (FES-I). *Age Ageing.* 2005;34(6):614-619.

8. Holden MK, Gill KM, Magliozzi MR, Nathan J, Piehl-Baker L. Clinical gait assessment in the neurologically impaired. Reliability and meaningfulness. *Phys Ther.* 1984;64(1):35-40.
