# Supplementary material for: The efficacy of a task model approach to ADL rehabilitation in stroke apraxia and action disorganisation syndrome: A randomised controlled trial
Source: PLoS One. 2022 Mar 3;17(3):e0264678. doi: 10.1371/journal.pone.0264678 (PMC8893688; doi:10.1371/journal.pone.0264678)
Supplement: S1 Text — (DOCX) [file pone.0264678.s003.docx]

***S1_Text***

***Supporting analyses***

The efficacy of a task model approach to ADL rehabilitation in stroke apraxia and action disorganisation syndrome: A randomised controlled trial.

Jo Howe^1,2^ ***^¶^***, Winnie Chua^1,3^ ***^¶^*** *, Emily Sumner^1^, Bogna Drozdowska^1^, Rosanna Laverick^1^, Rachel L Bevins^1,4^, Emilie Jean-Baptiste^5^, Martin Russell^5^, Pia Rotshtein^1^, Alan M Wing^1^.

^1^School of Psychology, College of Life and Environmental Sciences, University of Birmingham, Birmingham, UK.

^2^School of Pharmacy, Aston University, Birmingham, UK.

^3^Institute of Cardiovascular Sciences, College of Medical and Dental Sciences, University of Birmingham, Birmingham, UK.

^4^ School of Life Sciences, Faculty of Health and Life Sciences, Coventry University, Coventry, UK.

^5^School of Electronic, Electrical and Systems Engineering, College of Engineering and Physical Sciences, University of Birmingham, Birmingham, UK.

*Corresponding author

Winnie Chua

Email: [w.chua.1@bham.ac.uk](mailto:w.chua.1@bham.ac.uk) (WC)

***¶*** These authors contributed equally to this work.

***Results***

Of the 29 community dwelling stroke survivors recruited to participate in the study, 22 completed both training interventions and all assessments, and were included in the sensitivity analysis.

*Simple tea making*

The total number of errors across 22 participants making 2 cups of each of 4 types of tea on 4 assessments (a total of 704 cups) was 805, of which 527 were recoverable and 278 were non-recoverable. Inspection of the average number of errors (**Figure S1a**) and time taken (**Figure S1b**) across the 4 assessment sessions reveals reductions due to training that exceed the reductions in the control condition, in both Groups 1 and 2.

**
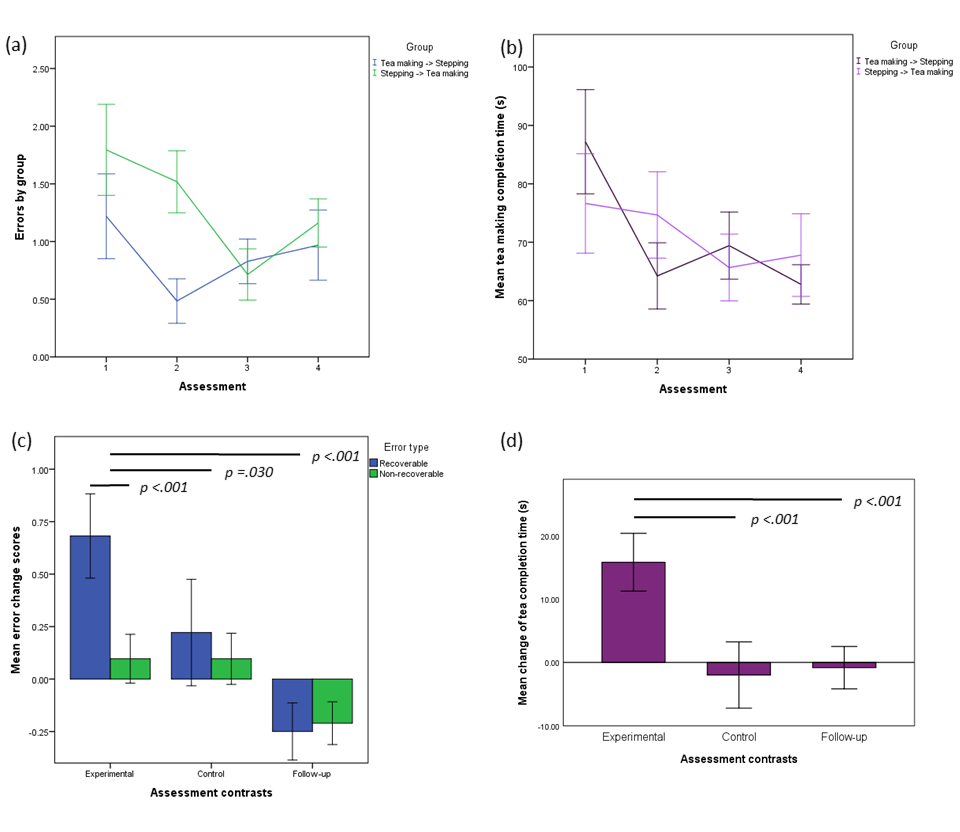
**

***Figure S1:*** ***Tea making****: (a) error (b) time taken for the two patient groups (Group 1: Baseline (1), Post-training (2), Post-control (3), Follow-up (4); Group 2: Baseline (1), Post-control (2), Post-training (3), Follow-up (4)). Tea making change scores (c) Recoverable and nonrecoverable errors (d) time as a function of condition. Experimental, control and follow-up scores were calculated 1-2, 2-3, 2-4 (where experimental, control and follow-up were calculated as Group1) and 2-3,1-2, 3-4 (Group 2). Statistically significant contrasts are shown across the top of (c,d).*

**Figures S1c and S1d** show the error (recoverable and non-recoverable) change scores and time change scores respectively, for experimental (before vs after tea making training), control (before vs after stepping training) and follow-up (post tea making training vs follow up assessment) contrasts (Group1 assessments 1-2, 2-3, 2-4 and Group 2 assessments 2-3, 1-2, 3-4) respectively.

2 x 3 repeated measures ANOVA was conducted with error type (recoverable errors, non-recoverable errors) and contrast (experimental, control, follow-up) as factors. There was a significant main effect of error type *F(*1,87) = 14.283, *p*<.001 and assessment condition *F*(1,172)=17.248, *p*<.001. There was also a significant interaction between error type and contrast *F*(1.836,159.771)=7.124, *p*=.002 (Greenhouse-Geisser correction, Ɛ=0.918).

Post-hoc pairwise comparisons indicated that there was a larger overall number of recoverable errors (mean±SE .218±.054) compared to non-recoverable (-.006±.025). The total number of errors reduced between experimental (.389±.057) and control contrasts (.159±.068; *p*=.030), between experimental and follow-up contrasts (-.230±.049; *p*<.001), as well as between control and follow-up contrasts (*p*<.001). The interaction effect was driven by differences between recoverable (.682±.100) and non-recoverable errors (.097±.058; *p*<.001) in the experimental contrast but not in the control (*p*=.392) and follow-up (*p*=.572) contrasts.

A one-way repeated measures ANOVA with Greenhouse-Geisser correction (χ^2^(2)=36.148, *p*<.001) indicated that mean change in time to complete tea making was significantly different between assessment contrasts (experimental, control, follow-up) *F*(1.486,129.545)=16.225, *p*<.001. Post-hoc pairwise comparisons revealed that time reduced between experimental (mean±SE 15.801±2.280 s) and control (-1.983±2.615 s; *p<*001) as well as experimental and follow-up (-.824±1.672 s; *p<*001) contrasts but there was no significant difference between control and follow-up contrasts (*p*=.690).

*Complex Tea making*

**Figure S2a** shows the number of errors in the untrained complex tea making task as a function of error type (recoverable vs non-recoverable) for the two groups (c.f. **Figure S1a**). This shows a reduction of errors in group 1 before and after simple tea making training (session 2 vs 1). However, group 2 also shows a reduction before and after the control condition and no reduction from session 2 to 3 associated with simple tea making training.

**
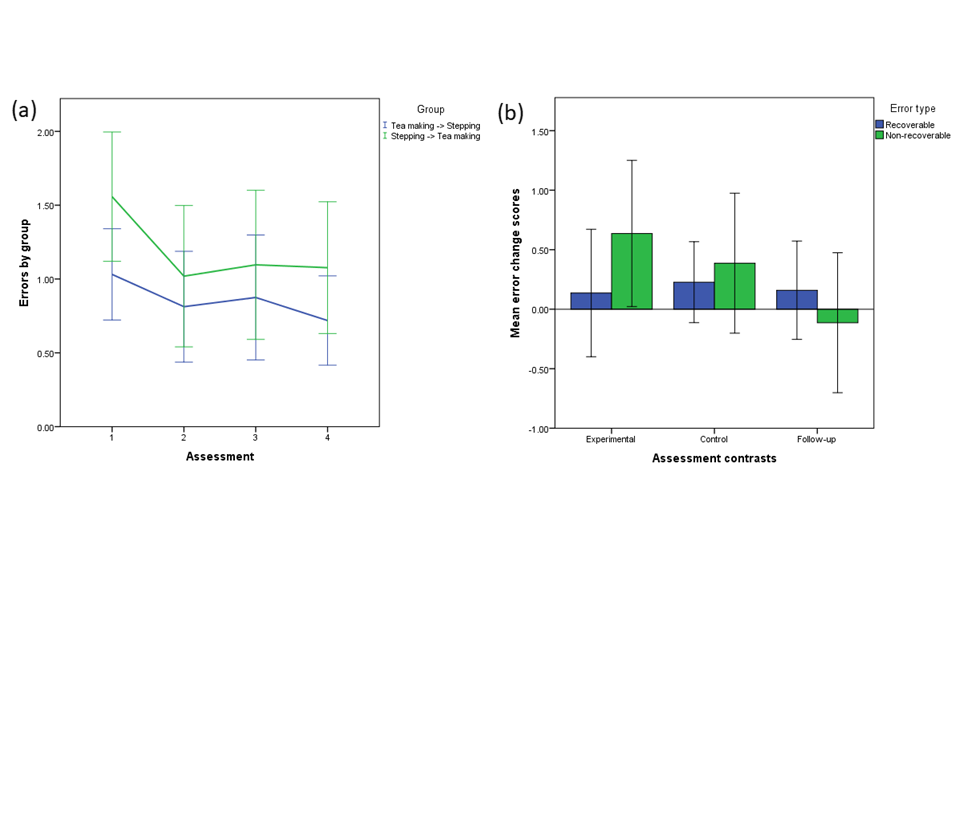
**

***Figure S2: Complex tea making:*** *(a) Proportion of complex tea making errors as a function of training condition. (b) Mean change in complex tea making errors across contrasts.*

**Figure S2b** shows the error (recoverable and non-recoverable) scores as a function of experimental (before vs after tea making training), control (before vs after stepping training) and follow-up (post tea making training vs follow up assessment) contrasts (Group1 assessments 1-2, 2-3, 2-4 and Group 2 assessments 2-3, 1-2, 3-4) respectively (c.f. Fig 2a). 2 x 3 repeated measures ANOVA was conducted with error type (recoverable errors, non-recoverable errors) and contrast (experimental, control, follow-up) as factors. There was no significant main effect of error type (p=.507) or assessment condition (p=.374; Greenhouse-Geisser correction).
