# Supplementary material for: The efficacy of a task model approach to ADL rehabilitation in stroke apraxia and action disorganisation syndrome: A randomised controlled trial
Source: PLoS One. 2022 Mar 3;17(3):e0264678. doi: 10.1371/journal.pone.0264678 (PMC8893688; doi:10.1371/journal.pone.0264678)
Supplement: S1 File — (PDF) [file pone.0264678.s004.pdf]

UNIVERSITY OF BIRMINGHAM
APPLICATION FOR ETHICAL REVIEW –
REQUEST FOR AMENDMENTS

Who should use this form:

- This form is to be completed by PIs or supervisors (for PGR student research) who are requesting ethical approval for amendments to research projects that have previously received ethical approval from the University of Birmingham.

  Please be aware that all new research projects undertaken by postgraduate research (PGR) students first registered as from 1st September 2008 will be subject to the University's Ethical Review Process. PGR students first registered before 1st September 2008 should refer to their Department/School/College for further advice.

- What constitutes an amendment?

  Amendments requiring approval may include, but are not limited to, additions to the research protocol, study population, recruitment of participants, access to personal records, research instruments, or participant information and consent documentation. Amendments must be approved before they are implemented.

NOTES:

- Answers to questions must be entered in the space provided
- An electronic version of the completed form should be submitted to the Research Ethics Officer, at the following email address: aer-ethics@contacts.bham.ac.uk. Please **do not** submit paper copies.
- If, in any section, you find that you have insufficient space, or you wish to supply additional material not specifically requested by the form, please submit it in a separate file, clearly marked and attached to the submission email.
- If you have any queries about the form, please address them to the Research Ethics Team.

**UNIVERSITY OF BIRMINGHAM<br>APPLICATION FOR ETHICAL REVIEW -<br>REQUEST FOR AMENDMENTS**	*OFFICE USE ONLY:*<br>Application No:<br>Date Received:

1. **TITLE OF PROJECT**

CogWatch - Cognitive rehabilitation of apraxia and action disorganisation

2. **APPROVAL DETAILS**

What is the Ethical Review Number (ERN) for the project?

ERN-12-0683

3. **THIS PROJECT IS:**

University of Birmingham Staff Research project **x**
University of Birmingham Postgraduate Research (PGR) student project **x**
Other ☐ (Please specify):

4. **INVESTIGATORS**

a) **PLEASE GIVE DETAILS OF THE PRINCIPAL INVESTIGATORS OR SUPERVISORS (FOR PGR STUDENT PROJECTS)**

Name: Title / first name / family name	Dr Pia Rotshtein
Highest qualification & position held:	PhD, Lecturer
School/Department	Psychology
Telephone:	0121 414 2879
Email address:	p.rotshtein@bham.ac.uk

Name: Title / first name / family name	Prof Alan Wing
Highest qualification & position held:	PhD, Chair
School/Department	Psychology
Telephone:	
Email address:	a.wing@bham.ac.uk

b) **PLEASE GIVE DETAILS OF ANY CO-INVESTIGATORS OR CO-SUPERVISORS (FOR PGR STUDENT PROJECTS)**

Name: Title / first name / family name	Prof Glyn W Humphreys
Highest qualification & position held:	PhD, Chair
School/Department	Psychology, Oxford University
Telephone:	
Email address:	Glyn.humphreys@oxford.ac.uk

c) **In the case of PGR student projects, please give details of the student**

Name of student:	Melanie Wulff	Student No:	1158625
Course of study:	PhD	Email address:	Mxw127@bham.ac.uk
Principal	Glyn Humphreys		
Name of student:	Amy Arnold	Student No:	1132022
Course of study:	PhD	Email address:	Axa052@bham.ac.uk
Principal supervisor:	Alan Wing		
Name of student:	Joanne Howe	Student No:	1302657
Course of study:	Master	Email address:	Jxh307@bham.ac.uk
Principal	Alan Wing		

5. **ESTIMATED START OF PROJECT** Date: 1-9-2012

ESTIMATED END OF PROJECT Date: 31-9-2016

6. **DETAILS OF PROPOSED AMENDMENTS**

We are at the prototype stage of the development of the COGWATCH system for tea making. The amendments requested in to enable to evaluate the rehabilitation efficacy of this prototype. To do that we would test patient ability to make a cup of tea before and after a training sessions with the system. A similar training protocol for CogWatch was approved by the NHS ethics 12/WM/02/20. In addition, we would include a control condition of lower gait training, training to walk and move the legs. The gait training protocol was approved by the NHS 10/H1207/28.

Main change from the original protocol is the requests to run multiple sessions with pre-selected patients. The changes have been in cooperated in an updated specific information sheet and a consent form (appendix 1), changes are highlighted in red.

Patient selection: based on initial screening only patients who show apraxia and action Disorganization (AAD) symptoms will be invited to take part in this training procedure. WE aim to recruit 30 patients from the stroke support clubs and the university patients' panel. The patients will be identified based on an initial screening procedure (already approved NHS 12/WM/02/20). Any patients who score below the cut offs for in at least one of following tasks:

Based Birmingham Cognitive Screen (BCoS, http://www.cognitionmatters.org.uk/)

1) Gesture production
2) Gesture recognition
3) Gesture imitation
4) Torch assembly

CogWatch screen tasks:

1) Spontaneous tea making
2) Complex tea making
3) filling

Multiple sessions procedure:

Pre-assessment –> training –> post-assessment 1 –> training –> post-assessment 2 –> follow up assessment.

Training sessions:

Mood assessment using the Hospital Anxiety and Depression Scales (HADS, appendix 2)

Cogwatch - the patients make cups of tea guided and monitored by the Cogwatch system.

Lower gait – the patients step in place or tap (if on a wheel chair) to a beat.

There would be four-five weekly training sessions of one type in each time window. The order of the training type (Cogwatch/Gait) would be counterbalanced across patients.

Assessment sessions:

All assessment sessions will be identical

Mood assessment using the HADS, blood pressure and heard rate using CogWatch developed device,

CogWatch – patients would repeat the CogWatch screening tasks and make cups of tea for specification (e.g. tea with no milk plus one sugar).

Lower limb gait – assessment will include for example: i) number of step/tap in place with no beat; ii) Fear of falling questionnaire (FES-I; appendix 3); iii) Fugl-Meyer test (http://www.rehabmeasures.org/lists/rehabmeasures/dispform.aspx?ID=908).

7. **JUSTIFICATION FOR AMENDMENTS**

As mentioned above, the CogWatch prototype for tea making is ready to be evaluated. To properly

assess its rehabilitation efficacy we design a pre-training-post study.<br>To insure that any improvement do not arise from simply repeating the assessment tasks twice, or from having regular contact with researcher we added a control condition, namely the lower gait training. The training gait protocol was chosen as the control condition, as lower limb gait (stable walking) is a frequent challenge after stroke. Hence this training is also likely to benefit patients.

8. **ETHICAL CONSIDERATIONS**

What ethical considerations, if any, are raised by the proposed amendments?

9. **DECLARATION BY APPLICANTS**

I make this application on the basis that the information it contains is confidential and will be used by the University of Birmingham for the purposes of ethical review and monitoring of the research project described herein, and to satisfy reporting requirements to regulatory bodies. The information will not be used for any other purpose without my prior consent.

I declare that:

- The information in this form together with any accompanying information is complete and correct to the best of my knowledge and belief and I take full responsibility for it.
- I undertake to abide by University Code of Conduct for Research (http://www.as.bham.ac.uk/legislation/docs/COP_Research.pdf) alongside any other relevant professional bodies' codes of conduct and/or ethical guidelines.
- I will report any changes affecting the ethical aspects of the project to the University of Birmingham Research Ethics Officer.
- I will report any adverse or unforeseen events which occur to the relevant Ethics Committee project to the University of Birmingham Research Ethics Officer.

**Signature of Principal investigator/project supervisor:**	Pia Rotshtein
**Date:**	13-5-2014

Appendix 1:
-
CogWatch: Cognitive rehabilitation of apraxia and action disorganisation
-
Participant information sheet and Consent form

CogWatch

UNIVERSITY OF BIRMINGHAM

Cognitive Rehabilitation of Apraxia & Action Disorganisation Syndrome

SyMon Lab, Hills Building
School of Psychology
University of Birmingham
Edgbaston, Birmingham B15 2TT
Tel: 0121 414 4932

Participants' Information Sheet: 'CogWatch - Cognitive rehabilitation of apraxia and action disorganisation'

You are receiving this letter because you have agreed to take part in this research following a phone conversation with Bogna Drozdowska (Participants coordinator) from the School of Psychology, University of Birmingham. We would first like to thank you for agreeing to help us with this research. Below you would find more information about the research, what it involves and the expected outcomes.

What is CogWatch?

CogWatch is a European Commission funded research project whose aim is to enhance the rehabilitation of stroke patients, a third of whom will experience long term physiological and/or cognitive disabilities.

A significant proportion of these patients can suffer from Apraxia or Action Disorganisation Syndrome (AADS) which, is characterised by an impairment of cognitive abilities to carry out activities of daily living (ADL).

CogWatch is co-ordinated by the University of Birmingham, and will develop advanced and intelligent, common objects and tools which will help to re-train patients in how to carry out ADL, by providing persistent multimodal feedback to them.

Who is conducting the research?

This research is conducted by a team of researchers from the School of Psychology, University of Birmingham in collaboration with researcher groups in Munich, Germany and Madrid, Spain. The Psychology Birmingham team is led by Prof Alan Wing, Prof Glyn Humphreys and Dr Pia Rotshtein. Research assistants within the school of psychology carry out the training sessions. The sessions are conducted in the Hills building.

What does the research involve?

The current research aims to evaluate how effective training is in assisting with the kinds of

activities you may be doing at home. The research is focussing on areas: tea making and stepping to music. You will be invited to attend the University for up to 14-17 sessions over a 5 month period. You will be assessed at various time points.. You will only do one activity at a time but we may ask you to do it multiple times, for example we will ask you to make a cup of tea several times in one visit. We expect that each session will last less than 1 ½ hours. You can have breaks whenever you need them.

Making cups of tea

For this part of the research you will be asked to make cups of tea in order that the effectiveness of an interactive computer based system that has been developed in assisting with this task can be evaluated.

Stepping to music

This part of the research will investigate whether there are benefits of stepping to a metronome in a weekly exercise programme based at the University. Previous research has indicated that hearing a cue such as a metronome during walking can have benefits for people post-stroke.

We will also use video cameras to records your actions and speech for later analyses. To protect your privacy the frames will not include your face, but only your torso and your hands.

If you have participated in research in the School of Psychology before, we will ask your permission to access previous data that was collected with you by research in the School. This specifically applies for brain imaging data behavioural data collected as part of the BCoS screen.

Are there any risks involved?

The tea making part of the research involves performing activities that you are likely to carry out routinely at home. Hence we do not anticipate that it would expose you to any risks beyond those which are expected in everyday life. The stepping part of the research involves walking and stepping so you might be tired afterwards. You will not be asked to do more than you want to and you can stop at any time. Specific breaks are provided, and you can request additional breaks at any time during each session. Furthermore, all measurements are non-invasive and do not pose any danger. The tea making part of the research involves boiling water in a kettle and pouring the water into a cup. In case you feel unsure about your ability to perform this task, we can provide a kettle tipper that restricts the pouring of the water. A research assistant will be present in the room throughout each session, monitoring for any unexpected accidents.

Why am I invited to participate in this research?

This research investigates the way people perform activities of daily leaving. We are interested to learn how who have suffered a stroke or from any other neurological condition perform these daily activities. Therefore you are invited either because you have a neurological condition.

Is the data anonymous?

Your personal details will be kept separately from the data in a locked file cabinet. You will be identified throughout the study using a random generated ID number. We will record your gender, age and health condition. However, as the sessions are being video recorded, it is impossible to keep the data completely anonymous, as the video will include information about your limbs, torso and possibly of your voice.

What will you do with my data?

The analysed data will be presented in scientific conferences and reported in scientific journals. The data collected in this research will further be used for the development of the CogWatch system. Therefore, it is likely that it will be shared with our research partners in Munich and Madrid. If you do not want your data to be shared with our partners, please insure you tick the appropriate box in the consent form to indicate that.

We may also present the data on our web page www.cogwatch.eu. If you do not want your data to be presented on the web page, please ensure that you tick the appropriate box in the consent form.

Would I be compensated for my travel?

We will arrange for a taxi to collect you from your home bring you to the University and return you home again. The University Of Birmingham will pay for this. Should you wish to travel in your own vehicle we will reimburse parking and mileage costs.

Can I withdraw from the study?

You can withdraw from the study, or parts of it, at anytime without the need to give any reason or justification. There will be no consequences for your withdrawal. If you decide to withdraw from the study, you would be compensated according to the time you spent doing the study till you withdraw. You can also ask to withdraw all your data, or part of your data up to one month after your participation. In this case your data or parts of your data based on your specification will be deleted from the data base.

What shall I do next?

We will arrange for a taxi to pick you up and bring you to the reception of the School of Psychology, at the Hills building, University of Birmingham. You may if you wish bring a carer with you If you decide that you are not interested to take part in this research please let us know.

For more details:

If you required any more details please feel free to contact us:

Ms. Bogna Drozdowska 0121 414 2870; b.a.drozdowska@bham.ac.uk
Dr. Pia Rotshtein 0121 414 2879; P.Rotshtein@bham.ac.uk

CogWatch

UNIVERSITY OF BIRMINGHAM

Cognitive Rehabilitation of Apraxia & Action Disorganisation Syndrome

SyMon Lab, Hills Building
School of Psychology
University of Birmingham
Edgbaston, Birmingham B15 2TT
Tel: 0121 414 4932

Consent form: 'The making of a cup of tea'

Name:_______________________________ **Date of Birth:** __________________________

Gender: Female / Male **Handedness: Right / Left**

	yes	No
I have read the information sheet		
I have received enough information about the study		
I had a chance to ask questions		
I have received satisfactory answers to my questions		
I understand that I am free to leave the study:<br>· at any time?<br>· without having to give a reason for leaving?<br>· without affecting my medical care?		
I agree that my hand movements and eye gaze be recorded		
I agree that hand and eye movements data will be shared with the research partners in Munich and Madrid.		
I agree that my hand and gaze movement data be presented on the cogwatch webpage and be made available to the general public.		
I agree that my session will be video taped		
I understand that the videos will include information that can identify me		
I agree that the data from the videos will be shared with the research partners in Munich and Madrid.		
I agree that my data from the videos be presented on the cogwatch webpage and be made available to the general public.		
If applicable, I agree that the current research will use brain-MRI and BCoS behavioural data previously collected from me at the School of Psychology		

Participant signature: ……………………………………..……………………..
Name of witness: ....
Witness signature: ....
Date: .... **Participants ID:** ____________________
