# Supplementary material for: The efficacy of a task model approach to ADL rehabilitation in stroke apraxia and action disorganisation syndrome: A randomised controlled trial
Source: PLoS One. 2022 Mar 3;17(3):e0264678. doi: 10.1371/journal.pone.0264678 (PMC8893688; doi:10.1371/journal.pone.0264678)
Supplement: S2 File — (PDF) [file pone.0264678.s005.pdf]

UNIVERSITY OF BIRMINGHAM
APPLICATION FOR ETHICAL REVIEW

Who should use this form:

This form is to be completed by PIs or supervisors (for PGR student research) who have completed the University of Birmingham's Ethical Review of Research Self Assessment Form (SAF) and have decided that further ethical review and approval is required before the commencement of a given Research Project.

Please be aware that all new research projects undertaken by postgraduate research (PGR) students first registered as from 1st September 2008 will be subject to the University's Ethical Review Process. PGR students first registered before 1st September 2008 should refer to their Department/School/College for further advice.

Researchers in the following categories are to use this form:

1. The project is to be conducted by:
    - staff of the University of Birmingham; or
    - a research postgraduate student enrolled at the University of Birmingham (to be completed by the student's supervisor);
2. The project is to be conducted at the University of Birmingham by visiting researchers.

Students undertaking undergraduate projects and taught postgraduates should refer to their Department/School for advice.

NOTES:

- Answers to questions must be entered in the space provided.
- An electronic version of the completed form should be submitted to the Research Ethics Officer, at the following email address: aer-ethics@contacts.bham.ac.uk. Please **do not** submit paper copies.
- If, in any section, you find that you have insufficient space, or you wish to supply additional material not specifically requested by the form, please it in a separate file, clearly marked and attached to the submission email.
- If you have any queries about the form, please address them to the Research Ethics Team.

X Before submitting, please tick this box to confirm that you have consulted and understood the following information and guidance and that you have taken it into account when completing your application:

- **The information and guidance provided on the University's ethics webpages (http://www.rcs.bham.ac.uk/ethics/index.shtml)**
- **The University's Code of Practice for Research (http://www.as.bham.ac.uk/legislation/docs/COP_Research.pdf)**

UNIVERSITY OF BIRMINGHAM APPLICATION FOR ETHICAL REVIEW	*OFFICE USE ONLY:* Application No: **ERN_12-0683** Date Received:

1. TITLE OF PROJECT

CogWatch - Cognitive rehabilitation of apraxia and action disorganisation

2. THIS PROJECT IS:

University of Birmingham Staff Research project **X**
University of Birmingham Postgraduate Research (PGR) Student project **X**
Other ☐ (Please specify):

3. INVESTIGATORS

a) PLEASE GIVE DETAILS OF THE PRINCIPAL INVESTIGATORS OR SUPERVISORS (FOR PGR STUDENT PROJECTS)

Name: Title / first name / family name	Dr Pia Rotshtein
Highest qualification & position held:	PhD, Lecturer
School/Department	Psychology
Telephone:	0121 414 2879
Email address:	p.rotshtein@bham.ac.uk

Name: Title / first name / family name	Prof Alan Wing
Highest qualification & position held:	PhD, Chair
School/Department	Psychology
Telephone:	
Email address:	a.wing@bham.ac.uk

b) PLEASE GIVE DETAILS OF ANY CO-INVESTIGATORS OR CO-SUPERVISORS (FOR PGR STUDENT PROJECTS)

Name: Title / first name / family name	Prof Glyn W Humphreys
Highest qualification & position held:	PhD, Chair
School/Department	Psychology, Oxford University
Telephone:	
Email address:	Glyn.humphreys@oxford.ac.uk

c) In the case of PGR student projects, please give details of the student

Name of student:	Melanie Wulff	Student No:	1158625
Course of study:	PhD	Email address:	Mxw127@bham.ac.uk
Principal	Glyn Humphreys		
Name of student:	Amy Arnold	Student No:	1132022
Course of study:	PhD	Email address:	Axa052@bham.ac.uk
Principal	Alan Wing		
Name of student:	Eva Fringi	Student No:	
Course of study:	Master	Email address:	Exf111@bham.ac.uk
Principal	Alan Wing		

4. ESTIMATED START OF PROJECT Date: 1-7-2012

ESTIMATED END OF PROJECT Date: 31-9-2016

5. FUNDING

List the funding sources (including internal sources) and give the status of each source.

Funding Body	*Approved/Pending /To be submitted*
FP7 CogWatch: A European collaborative projects. http://www.cogwatch.eu/	Approved

If applicable, please identify date within which the funding body requires acceptance of award:

Date:

If the funding body requires ethical review of the research proposal at application for funding please provide date of deadline for funding application:

Date:

6. SUMMARY OF PROJECT

Describe the purpose, background rationale for the proposed project, as well as the hypotheses/research questions to be examined and expected outcomes. This description should be in everyday language that is free from jargon. Please explain any technical terms or discipline-specific phrases.

After a stroke, patients can suffer from a wide range of problems depending on which area of their brain was affected. Physical impairments, such as problems with motor movements, vision or balance, are addressed with physical therapy but cognitive impairments, such as problems with language, memory or problem solving are harder to identify and may overlooked during a patient's rehabilitation. Though these later cognitive problem often have negative impact on the patients' well being.

'Apraxia and Action Disorganisation Syndrome' (AADS) is a common disorder following stroke. Patients who suffer from AADS have trouble performing ordered sequences of movements, such as those required to make a cup of tea or to brush their teeth. Even patients with normal movement of their hands and arms find themselves unable to complete everyday activities because they cannot execute the correct sequence of movements necessary to complete a task.

In the UK as many as 68% of stroke patients have problems typical of AADS (Bickeron et al., 2012). AADS can have a significant effect on a patient's recovery after stroke (Bickeron et al., 2012) and on their ability to live independent lives in their own homes.

The aim of the current project is to investigate the neuro-cognitive mechanisms that support the ability to complete activity of daily leaving such as making tea, making a toast, grooming etc. This information would then be used to develop the CogWatch system. The CogWatch will be based on a computer algorithm that monitors patients behaviour while executing daily life activity, and providing the patient with online feedback when an error is detected. The current research will record the behaviour of patients and healthy controls while performing everyday life activities.
Five types of daily activities will be tested (see **Appendix 1** for examples of the experimental set-up):

1) Making a cup of black tea
2) Making toast
3) Documents Filing
4) Assembling a torch
5) Complex tea making task: making two different cups of teas.

We would measure the time and the type of errors made when executing the above tasks. Furthermore, we would introduce various distractions and measure their effects on the overall task performances. The distraction would include: 1) Visual-Objects distracters, here not all items on the table are necessary for task completion (see **Appendix 1** for examples). 2) Two types of cognitive distractions: i) reciting sequences of numbers and letters while executing the task and ii) stopping the task sequence to complete a short calculation task on the computer.

7. CONDUCT OF PROJECT

Please give a description of the research methodology that will be used

Stimuli:
Real objects will be used in all tasks (see Appendix 1: for example of object display). The objects will be placed on a table in front of a sited participant.

Behavioural measures:

1) Motion capture: motion detectors would be attached to the objects and hand of the participants. The motion data will be recorded while the task is performed. The markers are small light spheres (~5mm). A Qualisys system (http://www.qualisys.com/) records the location of the markers using multiple cameras located around the room. These are not video cameras and they only capture information on the spatial location of the markers.
2) Body-motion capture: Kinect technology as implemented in x-box would be used to capture movements of the participants' limbs and torso in relation to the objects on the table. Kinect is not based on video cameras, but capture objects' movement in a 3D space and display it on cartoon avatars. This technology is commonly used in video games.
3) Eye tracking and first perspective video monitoring: A head mounted eye tracking system will be used (Diablis http://www.ergoneers.com/en/products/dlab-dikablis/testprocedure.html). Participants would wear a light elastic band on their forehead with an attached infra-red camera that record the location of their gaze and a video camera that record their visual field. The information of what they see and what they look at will be recorded.
4) Video recordings excluding the face. In case eye tracking will not be feasible we will video-record the session. The camera will be positioned such that the participant's face will not be included in the frame. The videos will include the view of the torso and the two hands as they operate on the items placed on the table.
5) A tick form: would be used by the experimenter for online monitoring of the steps taken when executing each behavioural task (see Appendix 2: example of scoring sheet).

Additional data: For the neurological patients we would collect data on the structure of their brain and their general cognitive profile. This data is available as part of the Bham patients' database. We would seek an approval from the patients to use that information.

Data analyses:
The data would be analysed using inbuilt analyses tools in each of the software and would be supported by home tailored Matlab scripts. Statistical analyses would be carried out using SPSS.
The following variables will be measured:

1) Time to complete each task
2) Time between each hand movement
3) The direction of gaze and the duration of dwelling time on each object.
4) The action sequence used in the task.

Based on our previous experience, we anticipate that healthy participants will complete all tasks within 2 hours, while some neurological patients will require 2 sessions of up to 2 hours for completing the tasks. Participants would be able to take breaks in between the tasks.

8. DOES THE PROJECT INVOLVE PARTICIPATION OF PEOPLE OTHER THAN THE RESEARCHERS AND SUPERVISORS?

Yes **X** No ☐

Note: "Participation" includes both active participation (such as when participants take part in an interview) and cases where participants take part in the study without their knowledge and consent at the time (for example, in crowd behaviour research).

If you have answered NO please go to Section 18 . If you have answered YES to this question please complete all the following sections.

9. PARTICIPANTS AS THE SUBJECTS OF THE RESEARCH

Describe the number of participants and important characteristics (such as age, gender, location, affiliation, level of fitness, intellectual ability etc.). Specify any inclusion/exclusion criteria to be used.

Participants: four group of participants will be tested:
Experimental group:
1) 30 Neurological patients who suffer from apraxia or action disorganization syndrome. These patients experience cognitive deficits that affect their ability to perform everyday tasks. Tasks that they were previously able to perform automatically. Diagnosis will be made based on their performance on the BCoS Aparxia section (Bickerton et a., 2012; www.bcos.bham.ac.uk)

Control groups:
2) 50 Young healthy participants age range 20-30y
3) 50 Elderly healthy participants – age range 60 – 80 year.
4) 30 Neurological patients who do not show problems typically associated with apraxia or action disorganization disorder.

10. RECRUITMENT

Please state clearly how the participants will be identified, approached and recruited. Include any relationship between the investigator(s) and participant(s) (e.g. instructor-student).

Note: Attach a copy of any poster(s), advertisement(s) or letter(s) to be used for recruitment.

Participants Recruitment
1) Young healthy participants will be recruited through: i) the research participation scheme and ii) advertisement in JobZone (http://www.guildofstudents.com/content/index.php?page=29305).
2) Elderly healthy participants will be recruited from: i) the School of Psychology's database (Bham Panel) for volunteer participants and ii) through recruitment posters that would be presented on public notice boards. (see Appendix 3: recruitment posters)
3) Neurological patients will be recruited from: i) the School of Psychology's database of patients (Bham Panel). These patients volunteer on a regular base to take part in experiments run in the school; ii) through announcements and posters distributed by the stroke association (see Appendix 3: recruitment posters), iii) from the BUCS database, and iv) from the neurological department in Mosley hall and the Queen Elizabeth hospital. We have submitted a separate ethics for the NHS for the approval of the recruitments of patients form the BUCS and the hospital wards. These later patients would not be recruited until approval would be granted by the NHS appropriate committee.

11. CONSENT

a) Describe the process that the investigator(s) will be using to obtain valid consent. If consent is not to be obtained explain why**.** If the participants are minors or for other reasons are not competent to consent, describe the proposed alternate source of consent, including any permission / information letter to be provided to the person(s) providing the consent.

Consent is obtained in two steps:
1) Patients and elderly controls form the Bham panel. Initial phone contact with the participant is made by Denise Clissett who is the participant coordinator of the panel. She will provide initial introduction of the study. She would explain that we are recruiting participants for experiments that investigate the ability to perform everyday life activity, such as making tea. If the participants (healthy or patients) are interested in taking part, she would schedule a meeting for them. In addition she would post for them the study information sheet (see Appendix 4).

At the beginning of the experimental session the experimenter will present the information sheet to all participants (including the young healthy) and would discussed it with them.

Note: Attach a copy of the Participant Information Sheet (if applicable), the Consent Form (if applicable), the content of any telephone script (if applicable) and any other material that will be used in the consent process.

b) Will the participants be deceived in any way about the purpose of the study? **Yes** ☐ **No x**

If yes, please describe the nature and extent of the deception involved. Include how and when the deception will be revealed, and who will administer this feedback.

12. PARTICIPANT FEEDBACK

Explain what feedback/ information will be provided to the participants after participation in the research. (For example, a more complete description of the purpose of the research, or access to the results of the research).

At the end of the experiment participants would be debriefed. They would be asked the following questions: 1) how did they felt during the experiment, 2) whether the recording equipment and the monitor of their behaviour was uncomfortable in anyway, and 3) how do they think they preformed. They would receive verbal feedback on their accuracy and if needed their errors would be explained to them. In addition they would be directed to the project web site if they are interested in following up the progress of the project.

13. PARTICIPANT WITHDRAWAL

a) Describe how the participants will be informed of their right to withdraw from the project.

Participants would be informed that they can withdraw at anytime from the experiment. They would also be able to withdraw only from parts of the study, and asked that some of the information would not be recorded (for example, if they do not wish to be filmed).

b) Explain any consequences for the participant of withdrawing from the study and indicate what will be done with the participant's data if they withdraw.

If a participant asks to withdraw or partly withdraw, then they will be asked if they want their data to be deleted from the study. We would delete their data, if the participants ask for it. There will be no consequences for withdrawal.

14. COMPENSATION

Will participants receive compensation for participation?

i) Financial **Yes x No** ☐

ii) Non-financial **Yes x No** ☐

If **Yes** to **either** i) or ii) above, please provide details.

Compensation:
All participants would be offered £7 per hour of participation. Taxi service will be used to bring the patients to the University.

If participants choose to withdraw, how will you deal with compensation?

Participants will be compensated for the time they have spent till they withdraw and/or for their travel expenses if needed.

15. CONFIDENTIALITY

a) Will all participants be anonymous? **Yes** ☐ **No x**
b) Will all data be treated as confidential? **Yes x** **No** ☐

Note: Participants' identity/data will be confidential if an assigned ID code or number is used, but it will not be anonymous. Anonymous data cannot be traced back to an individual participant.

Describe the procedures to be used to ensure anonymity of participants and/or confidentiality of data both during the conduct of the research and in the release of its findings.

All participants will be given an ID number, and would be identified throughout the study using this number. The indexing of personal details, consent forms and ID numbers will be kept separately from the data in a lock file cabinet.

If participant anonymity or confidentiality is not appropriate to this research project, explain, providing details of how all participants will be advised of the fact that data will not be anonymous or confidential.

1) The data include videos of the participants performing the tasks. Hence the data cannot be completely anonymous. Participants would be informed that their performances would be recorded in video (see Appendix 4), and would be explicitly asked to agree to that in the consent forms (Appendix 5).
2) The data may be shared with our European collaborators. Note that the participants would be notified of that in the information sheet (Appendix 4) and would explicitly asked to agree for the sharing of their data with our European collaborators in the consent form (Appendix 5).
3) Furthermore we may present part of the individual data on the project web page, again participants would be explicitly asked for permission to do so (Appendix 5).

16. STORAGE, ACCESS AND DISPOSAL OF DATA

Describe what research data will be stored, where, for what period of time, the measures that will be put in place to ensure security of the data, who will have access to the data, and the method and timing of disposal of the data.

The data would be stored in password locked computers placed within the SyMon lab. The data will be accessed primarily by researchers in the University of Birmingham that are involved in the CogWatch project. At the end of the project the data would be backed-up to a hard drive and kept for 10years.
As mention above, our research collaborators may be given access to some of the data. As the essence of this project is in the collaborations of different European research Centres, in which each centre contributes their expertise to the overall project.
The partners that will be given access to the data are:
1) Professor Joachim Hermsdörfer, Technische Universitat Munich. TUM's partners are running a parallel study in Germany, using an identical design. The data will be combined across centres to increase the overall study power.
2) Professor Manuel Ferre, Universidad Politecnica de Madrid. UPM's partners are responsible for developing the engineering part of the CogWatch system and would be using the data to simulate the system.

17. OTHER APPROVALS REQUIRED? e.g. Criminal Records Bureau (CRB) checks

☐ YES ☐ NO x NOT APPLICABLE

If yes, please specify.

18. SIGNIFICANCE/BENEFITS

Outline the potential significance and/or benefits of the research

The current research project would provide initial data to support the development of the CogWatch system. Cogwatch aims to help and provide support for the rehabilitation of AADS patients, enabling them to regain their ability to perform activities of daily living. The current project will provide both normative data on parameters that characterised normal performances across ages of everyday life activities and the type of errors that patients are likely to make.
In addition, it would facilitate patient assessment. There is no current systematic assessment and classification of AADS patients, as there is no reliable data available. We would provide systematic assessment and classification of AADS patients in two European countries, UK and Germany. The classification will result in different patient categories according to the severity of neurophysiological (e.g., identification of brain areas affected) and cognitive impairments (e.g., identification of affected ADL tasks).
The perceptual and cognitive distraction manipulations would enable to test different hypotheses on the sources of AADS. Specifically, are different types of AADS associated with differential consequences of stroke such as increase of perceptual load (the introduction of object distracters) or cognitive load (introduction of secondary cognitive task) due to deficits in processing resources.

19. RISKS

a) Outline any potential risks to **INDIVIDUALS**, including research staff, research participants, other individuals not involved in the research and the measures that will be taken to minimise any risks and the procedures to be adopted in the event of mishap

There are minimal risks involved in this research as all data collecting methods are non-invasive. Participants are asked to performed tasks with objects that they are likely to encounter in their everyday life.
The tea making tasks involves pouring boiling water into a cup of tea. There is a risk of self burn if the patient fails to pour the water into the cup. To minimize this risk, we will take the following actions:
1) We will limit the amount of water in the kettle to insure it is only contained what needed for 1-2 cups of tea.
2) We will assess the participant's ability to pour cold water correctly into a cup. If the participant fails or struggles with this task, we will replace the normal kettle with a specially designed kettle tipper. This makes the kettle easier to use, and the pouring of water is restricted in space, ensuring it will only fall into the cup.
3) An experienced experimenter will be constantly present in the room to monitor for any accidents.

Some patients may become frustrated, if failing to complete the tasks adequately. In such cases the experimenter and a more senior member of the research team will meet with the participant and would discuss in more details the aim of the research and how it may help to support their rehabilitation process. The patient would be asked again if s/he is willing to continue with the study and would be offered to come to additional sessions for further trainings.

b) Outline any potential risks to **THE ENVIRONMENT and/or SOCIETY** and the measures that will be taken to minimise any risks and the procedures to be adopted in the event of mishap.

NA

20. ARE THERE ANY OTHER ETHICAL ISSUES RAISED BY THE RESEARCH?

Yes ☐ No x

If yes, please specify

21. CHECKLIST

Please mark if the study involves any of the following:

- Vulnerable groups, such as children and young people aged under 18 years, those with learning disability, or cognitive impairments **x**
- Research that induces or results in or causes anxiety, stress, pain or physical discomfort, or poses a risk of harm to participants (which is more than is expected from everyday life) ☐
- Risk to the personal safety of the researcher ☐
- Deception or research that is conducted without full and informed consent of the participants at time study is carried out ☐
- Administration of a chemical agent or vaccines or other substances (including vitamins or food substances) to human participants. ☐
- Production and/or use of genetically modified plants or microbes ☐
- Results that may have an adverse impact on the environment or food safety ☐
- Results that may be used to develop chemical or biological weapons ☐

Please check that the following documents are attached to your application.

	ATTACHED	NOT APPLICABLE
Recruitment advertisement	X	☐
Participant information sheet	X	☐
Consent form	X	☐
Questionnaire	☐	☐
Interview Schedule	☐	☐
Experimental protocols	X	

22. DECLARATION BY APPLICANTS

I submit this application on the basis that the information it contains is confidential and will be used by the University of Birmingham for the purposes of ethical review and monitoring of the research project described herein, and to satisfy reporting requirements to regulatory bodies. The information will not be used for any other purpose without my prior consent.

I declare that:

- The information in this form together with any accompanying information is complete and correct to the best of my knowledge and belief and I take full responsibility for it.
- I undertake to abide by University Code of Practice for Research (http://www.as.bham.ac.uk/legislation/docs/COP_Research.pdf) alongside any other relevant professional bodies' codes of conduct and/or ethical guidelines.
- I will report any changes affecting the ethical aspects of the project to the University of Birmingham Research Ethics Officer.
- I will report any adverse or unforeseen events which occur to the relevant Ethics Committee via the University of Birmingham Research Ethics Officer.

**Name of Principal investigator/project supervisor:**	Pia Rotshtein
**Date:**	5/6/2012

Please now save your completed form, print a copy for your records, and then email a copy to the Research Ethics Officer, at aer-ethics@contacts.bham.ac.uk. As noted above, please do not submit a paper copy.

Appendix 1: experimental protocol

Tea preparation

Standard Task

Target objects

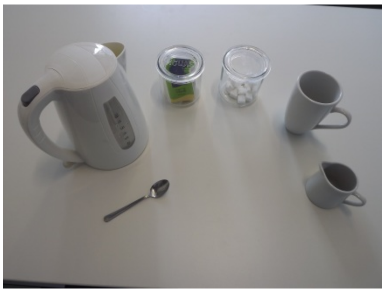

- Electric kettle
- Tea spoon
- Mug
- Jar of tea bags
- Milk jug
- Sugar jar

Instructions
1. Arrange the objects as shown above
2. Show the picture of the prepared cup of tea
3. Say to the participant: "*Can you please make a cup of tea. Everything you need is here for you. Do the best you can.*"
4. If after 30 sec., the patient fails to initiate any given action, then repeat the instruction and show the picture.
5. STOP if the patient still FAILS TO INITIATE any given step.

Tea preparation

Task with distractor objects

Target objects
- Electric kettle
- Tea spoon
- Mug
- Jar of tea bags
- Milk jug
- Sugar jar

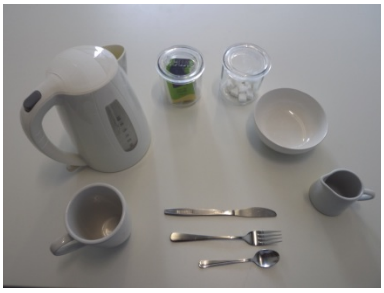

Distractor objects
- Fork
- Knife
- Cereal bowl

Instructions
1. Arrange the objects as shown above
2. Show the picture of the prepared cup of tea
3. Say to the participant: "*Can you please make a cup of tea. Everything you need is here for you. Do the best you can.*"
4. If after 30 sec., the patient fails to initiate any given action, then repeat the instruction and show the picture.
5. STOP if the patient still FAILS TO INITIATE any given step.

Document Filing

Standard Task

Target objects
- 2 sheets of paper
- Folder
- Stapler
- Hole punch

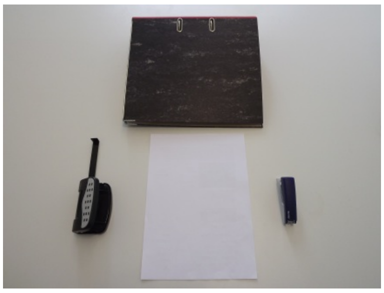

Instructions
1. Arrange the objects as shown above
2. Show the picture of the filed documents
3. Say to the participant: "*Can you staple the paper together and place the paper in the folder? Everything you need is here for you. Do the best you can*."
4. If after 30 sec., the patient fails to initiate any given action, then repeat the instruction and show the picture.
5. STOP if the patient still FAILS TO INITIATE any given step.

Document Filing

Task with distractor objects

Target objects
- 2 sheets of paper
- Folder
- Stapler
- Hole punch

Distractor objects
- Pen
- Gluestick
- Tape

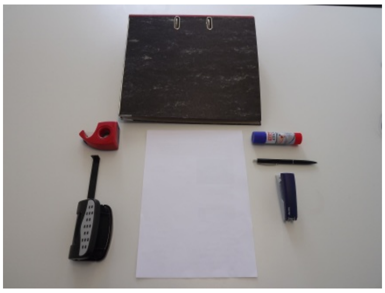

Instructions
1. Arrange the objects as shown above
2. Show the picture of the filed documents
3. Say to the participant: "*Can you staple the paper together and place the paper in the folder? Everything you need is here for you. Do the best you can*."
4. If after 30 sec., the patient fails to initiate any given action, then repeat the instruction and show the picture.
5. STOP if the patient still FAILS TO INITIATE any given step.

Toast making

Standard Task

Target objects

- Toaster
- Plate
- Knife
- Bread
- Butter
- Jam

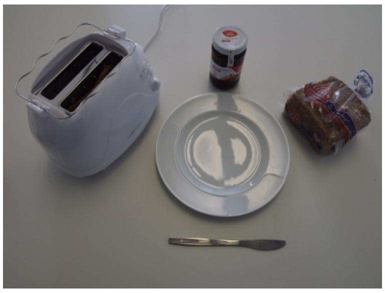

Instructions

1. Arrange the objects as shown above
2. Show the picture of the prepared toast
3. Say to the participant: "*Can you prepare a piece of toast with jam? Everything you need is here for you. Do the best you can*."
4. If after 30 sec., the patient fails to initiate any given action, then repeat the instruction and show the picture.
5. STOP if the patient still FAILS TO INITIATE any given step.

Toast making

Task with distractor objects

Target objects

- Toaster
- Plate
- Knife
- Bread
- Butter
- Jam

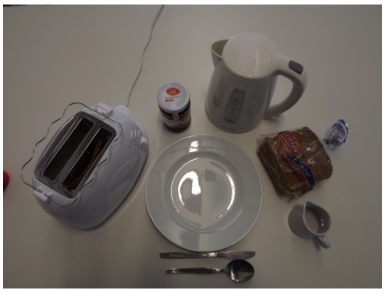

Distractor objects

- Spoon
- Milk
- Kettle

Instructions

1. Arrange the objects as shown above
2. Show the picture of the prepared toast
3. Say to the participant: "*Can you prepare a piece of toast with jam? Everything you need is here for you. Do the best you can*."
4. If after 30 sec., the patient fails to initiate any given action, then repeat the instruction and show the picture.
5. STOP if the patient still FAILS TO INITIATE any given step.

Complex tea-making task

Target objects

- Electric kettle
- Teaspoon
- Mug
- Transparent glass
- Jug of water
- Jar of tea bags
- Slices of lemon
- Jug of milk
- Jar of sugar cubes
- Sweetener
- Saucer for used tea bags

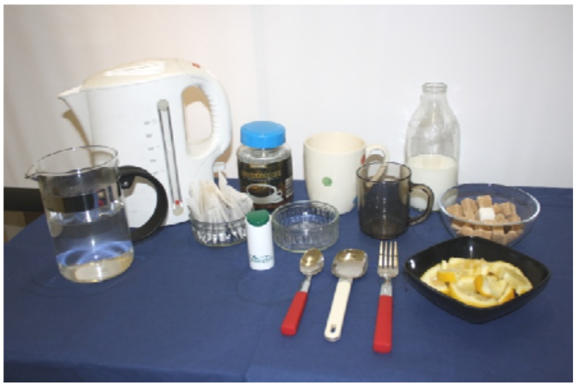

Distractor objects

- Dessert spoon
- fork
- Jar of coffee

Instructions

1. Arrange the objects as shown above
2. Provide the patient with the following *verbal* instructions: "Please can you make two cups of tea? One cup should be made with milk and two sweeteners and the other should be made with a slice of lemon and one sugar cube. Everything you need is here for you. Do the best you can.
3. If, after 30 seconds, the patient fails to initiate any given action then repeat the instruction *and* show the picture of the prepared cups of tea.
4. STOP if the patient FAILS TO INITIATE any given step
5. The patient should complete 2 trials of the complex tea-making task. If the patient fails to initiate the task using the verbal instructions alone they may still complete 2 further trials using both verbal and pictorial instructions.
6. Scoring for the complex tea-making task is based on the multi-step object use task (BCoS).

Picture of final goal: 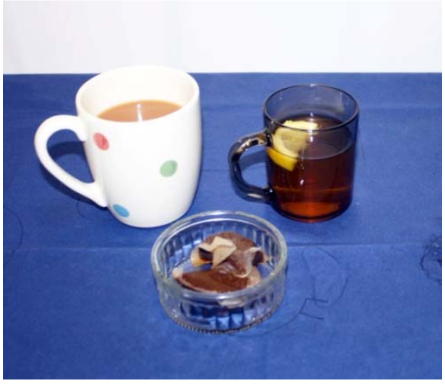

Please note: Water should be poured from the jug into the kettle. The jug should be filled with marginally more water than would be needed for 2 cups of tea. *The kettle should be placed in a safety tipper and the position of the mug/cup for safe pouring should be marked on the table and pointed out to the participant.*

Other completed Task Pictures

Torch

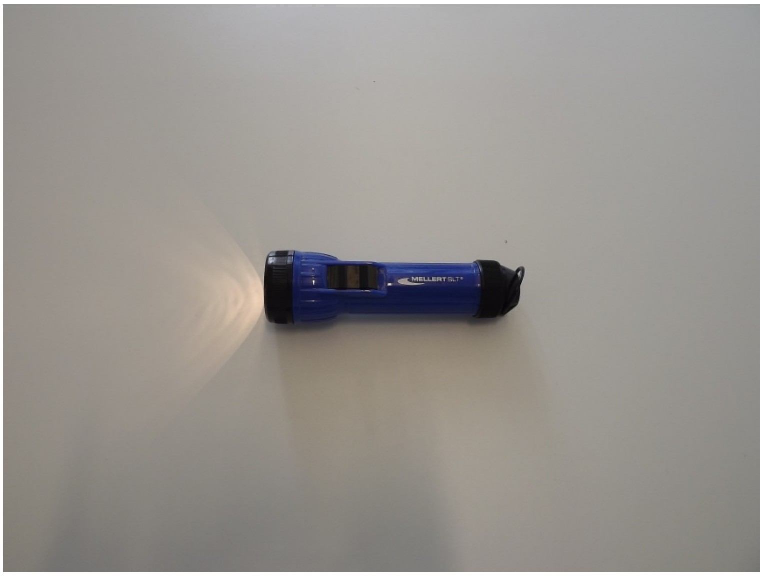

Cup of Tea

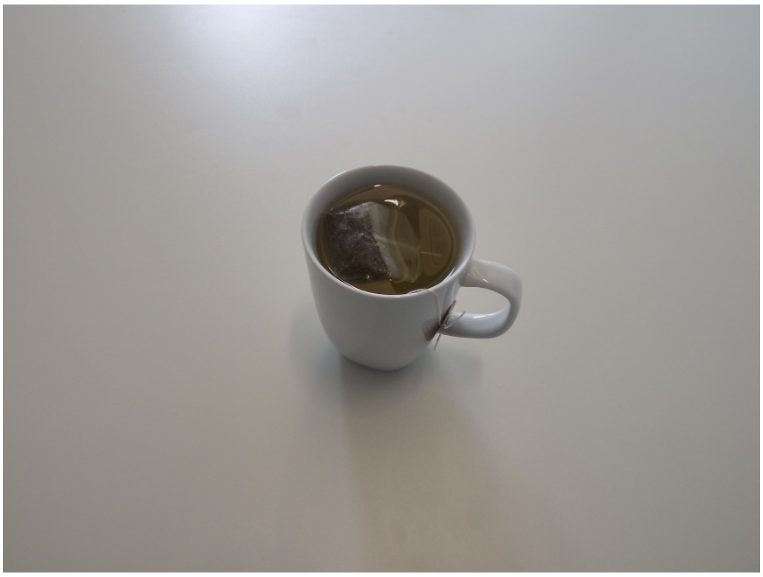

Filed documents

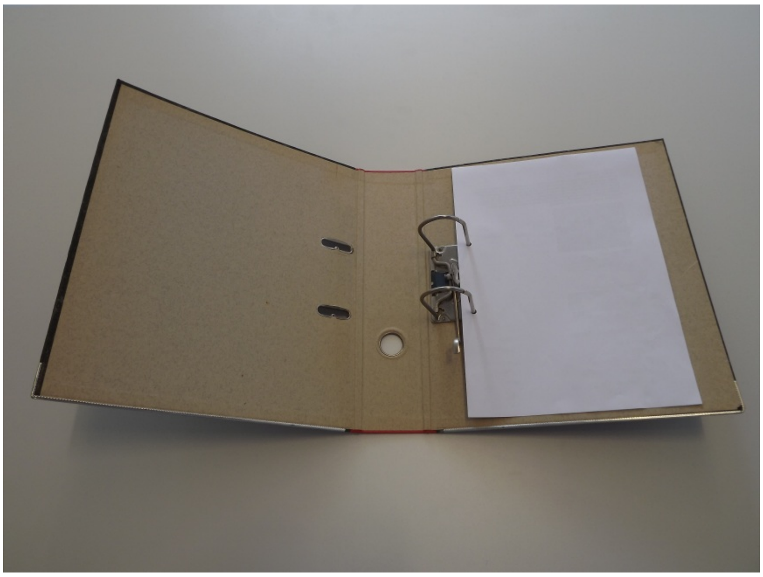

Toast preparation

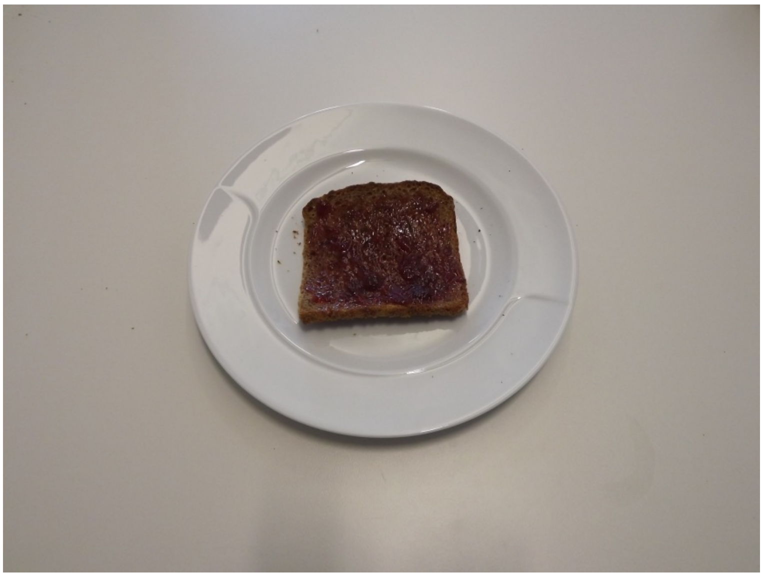

Appendix 2: Example of scoring sheet for complex tea making task

Please note: Water should be poured from the jug into the kettle. The jug should be filled with marginally more water than would be needed for 2 cups of tea. *The kettle should be placed in a safety tipper and the position of the mug/cup for safe pouring should be marked on the table and pointed out to the participant.*

Scoring:

SEQUENCE 1: **Tea & milk**	Order	Description
Heat water		
Place tea bag in the cup		
Add water in the cup		
Add sweeteners		
Add milk		
Remove teabag from cup		

SEQUENCE 2: **Tea & lemon**	Order	Description
Heat water		
Place tea bag in the cup		
Add water in the cup		
Add sugar		
Add lemon		
Remove teabag from cup		

Other:

Give 1 point for each criterion achieved on first attempt.

Fill kettle with jug of water	0 point	1 point
Switch on kettle, wait for boiling	0 point	1 point
Place teabag in cup	0 point	1 point
Pour water into cup	0 point	1 point
Put two sweeteners into cup	0 point	1 point
Pour **milk** into cup	0 point	1 point
Stir tea with spoon	0 point	1 point
Remove teabag	0 point	1 point
No use of irrelevant objects	0 point	1 point
No irrelevant actions with the target objects	0 point	1 point
No perseveration	0 point	1 point

Pick another cup	0 point	1 point
Place teabag in cup	0 point	1 point
Pour water into cup	0 point	1 point
Put only one sugar into cup	0 point	1 point
Put **lemon** into cup	0 point	1 point
Stir tea with spoon	0 point	1 point
Remove teabag	0 point	1 point
No use of irrelevant objects	0 point	1 point
No irrelevant actions with the target objects	0 point	1 point
No perseveration	0 point	1 point

Hand used: _____________
(B = both; L = left; R = right)
Condition of testing: _____________
(1=normal;
NT or stopped due to 2=aphasia; 3=visual/spatial; 4=confusion; 5=fatigue; 6=motor; 7=other.....)

Appendix 3: recruitment posters

A) To be place on notice boards

CogWatch

Cognitive
Rehabilitation of
Apraxia & Action
Disorganisation
Syndrome

The problem

Many stroke survivors suffer from **problems with mental processes** such as language, attention and memory.

The **CogWatch** project aims to help stroke patients who have trouble performing **ordered sequences of movements**, such as those required to make a cup of tea. These patients are diagnosed as suffering from apraxia and action disorganization syndrome (AADS)

How will CogWatch help?

The **CogWatch** researchers are investigating the specific problems faced by AADS patients and developing **new technologies** to assist them with their daily activities.

If **CogWatch** is successful it has the potential to:

- enable stroke patients with AADS to overcome the mental challenges that impair their daily lives
- improve their quality of life in the long term.

Do you want to help?

We are looking for young and old healthy volunteers to participate in our research. The research will involve completion of everyday task, like making a cup of tea.

If you would like more information, please contact:

Denise Clissett at the University of Birmingham.

Phone: 0121 414 4932; Email: D.Clissett@bham.ac.uk

B) To be distributed by the stroke association

CogWatch

CogWatch

Developing rehabilitation tools for stroke survivors with mental difficulties

The problem

Many stroke survivors suffer from **problems with mental processes** such as language, attention and memory. These difficulties are harder to identify than the physical symptoms of stroke and often get overlooked during a patient's rehabilitation.

Mental difficulties can have a very **negative impact** on a stroke survivor's quality of life and can increase their **dependence** on family members for daily support.

The CogWatch project aims to help stroke patients who have trouble performing **ordered sequences of movements**, such as those required to make a cup of tea or to brush their teeth. These patients may have normal movement of their hands and arms but struggle to complete everyday activities because they cannot execute the correct sequence of movements necessary to complete a task.

This type of impairment is termed 'Apraxia and Action Disorganisation Syndrome' (AADS) by doctors and, although it is hard to diagnose, it is quite common. Recently, scientists in the UK found that perhaps as many as 68% of stroke patients have problems typical of AADS.

How will CogWatch help?

The CogWatch researchers are investigating the specific problems faced by AADS patients and developing **new technologies** to assist them with their daily activities.

The ultimate aim is to develop a personalised rehabilitation system that can be installed into the homes of stroke survivors. It will silently monitor them as they go about their daily routine and provide helpful and relevant [illegible] em when they make errors.

CogWatch

Cognitive Rehabilitation of Apraxia & Action Disorganisation Syndrome

If CogWatch is successful it has the potential to:

- **enable stroke patients with AADS to overcome the mental challenges that impair their daily lives**
- **improve their quality of life in the long term.**

How will CogWatch work?

The system will use 'intelligent' everyday objects, like cutlery or a tea kettle, that contain sensors to monitor orientation, motion and grip strength. A central processing system will wirelessly collect the object data and combine it to assess how the objects are being held and used.

During a task, such as making a cup of tea, the system will track the actions of the user through the intelligent tools. When an error is detected, it will notify the user and provide guidance to assist them in completing the task.

Guidance could be in the form of relevant images on a display screen, audible sounds or instructions, or the physical vibration of a wrist watch.

The CogWatch system will:

- **Guide user actions to help complete daily tasks.**
- **Make users more aware of the mental errors they commit.**
- **Help users learn to overcome their errors.**
- **Alert users if their safety is at risk**

Do you want to help?

We are looking for stroke survivors who experience problems with completing everyday tasks to participate in our research.
If you live in the West Midlands area and would like to move information, please contact:

Denise Clissett at the University of Birmingham.
Phone: 0121 414 4932
Email: D.Clissett@bham.ac.uk

Appendix 4: **Study Information sheet**

CogWatch

UNIVERSITY OF BIRMINGHAM

Cognitiv
Rehabilitation c
Apraxia & Actio
Disorganisatio
Syndrom

SyMon Lab, Hills Building
School of Psychology
University of Birmingham
Edgbaston, Birmingham B15 2TT
Tel: 0121 414 4932

Participants' Information Sheet: 'Making of a cup of tea'

You are receiving this letter because you have agreed to take part in this research following a phone conversation with Denise Clissett (Participants coordinator) from the School of Psychology, University of Birmingham. We would first like to thank you for agreeing to help us with this research. Below you would find more information about the research, what it involves and the expected outcomes.

What is CogWatch?

CogWatch is a European Commission funded research project whose aim is to enhance the rehabilitation of stroke patients, a third of whom will experience long term physiological and/or cognitive disabilities.

A significant proportion of these patients can suffer from Apraxia or Action Disorganisation Syndrome (AADS) which, , is characterised by an impairment of cognitive abilities to carry out activities of daily living (ADL).

CogWatch is co-ordinated by the University of Birmingham, and will develop advanced and intelligent, common objects and tools which will help to re-train patients in how to carry out ADL, by providing persistent multimodal feedback to them.

Who is conducting the research?

This research is conducted by a team of researchers from the School of Psychology, University of Birmingham in collaboration with researcher groups in Munich, Germany and Madrid, Spain. The Psychology Birmingham team is led by Prof Alan Wing, Prof Glyn Humphreys and Dr Pia Rotshtein. The actual experiments would be carried out by Amy Arnold,a PhD student and Eva Fringi, a Masters student. The research would be conducted in the SyMon lab located in the Hills building.

What does the research involve?

The current research aims to understand how we performed activities of daily living. The research would focus on the following activities: making a cup of tea, making a toast, filing documents and assembling a torch. As you do these tasks we will monitor your hand movements and will track your eye gaze.

The hand movements will be monitored by attaching small (approx. 5mm sphere) markers to your hands. Special cameras will then be used to track the location of these markers in space, as you move your hands. We may also use Kinect as implemented in X-box (common used for video games) to track your movement in space. This is a special camera (not a video camera) that record the way you move in space and interacts with objects, it only record the movements and project them on an avatar body.

Eye gaze is tracked by wearing a light band over your forehead. This band holds a small camera that records the reflections of your cornea. The cornea's reflection indicates the direction of the gaze. This eye tracking device works well even if you wear glasses or contact lenses.

We will also use video cameras to records your actions and speech for a later analyses. To protect your privacy the frames will not include your face, but only your torso and your hands.

We will ask you to do these tasks multiple times. We expect that the entire sessions will last less than 2 hours. You can have breaks between the tasks. In case we are not able to collect all the data we need within 2 hours, we may ask you to come for another session on another day.

If you have participated in research in the School of Psychology before, we will ask your permeation to access pervious data that was collected with you by research in the School. This specifically applies for brain imaging data behavioural data collected as part of the BCoS screen.

Are there any risks involved?

The experiment involves performing activities that you are likely to carry out routinely at home. Hence we do not anticipate that it would expose you to any risks beyond those which are expected in everyday life. Furthermore, all measurements are non-invasive and do not pose any danger. Tea making involves boiling water in a kettle and pouring the water into a cup. In case you feel unsure about your ability to perform this task, we can provide a kettle tipper that restricts the pouring of the water. An experimenter will be present in the room throughout the experiment, monitoring for any unexpected accidents.

Why am I invited to participate in this research?

This research investigates the way people perform activities of daily leaving. We are interested to

learn how healthy participants perform these activities and how patients who suffered a stroke or from any other neurological condition performs these daily activities. Therefore you are invited either because you have a neurological condition or because you are neurologically healthy.

Is the data anonymous?

Your personal details will be kept separately from the data in a locked file cabinet. You will be identified throughout the study using a random generated ID number. We willrecord your gender, age and health condition. However, as the sessions are being video recorded, it is impossible to keep the data completely anonymous, as the video will include information about your limbs, torso and possibly of your voice.

What will you do with my data?

The analysed data will be presented in scientific conferences and reported in scientific journals. The data collected in this research will further be used for the development of the CogWatch system. Therefore, it is likely that it will be shared with our research partners in Munich and Madrid. If you do not want your data to be shared with our partners, please insure you tick the appropriate box in the consent form to indicate that.

We may also present the data on our web page www.cogwatch.eu. Again if you do not want your individual data be presented on the web page, please ensure that you tick the appropriate box in the consent form.

Would I be compensated for my time?

You would receive £7 per hour to compensate you for the time you spent participated in the study.

Can I withdraw from the study?

You can withdraw from the study, or parts of it, at anytime without the need to give any reason or justification. There will be no consequences for your withdrawal. You can also ask to withdraw all your data, or part of your data at any time before the project is completed. If you decide to withdraw, you would be compensated according to the time you spent doing the study till you withdraw.

What shall I do next?

Your scheduled appointment is on the ______________________________________.

Please arrive to the reception of the School of Psychology, at the Hills building, University of Birmingham. If you decide that you are not interested to take part in this research please let us know as soon as possible.

For more details:

If you required any more details please feel free to contact us:

Ms. Denise Clissett 0121 414 4932; D.Clissett@bham.ac.uk

Dr. Pia Rotshtein 0121 414 2879; P.Rotshtein@bham.ac.uk

Appendix 5: Consent form

CogWatch

UNIVERSITY OF BIRMINGHAM

SyMon Lab, Hills Building
School of Psychology
University of Birmingham
Edgbaston, Birmingham B15 2TT
Tel: 0121 414 4932

Cognitiv
Rehabilitation c
Apraxia & Actio
Disorganisatio
Syndrom

Consent form: 'The making of a cup of tea'

Name:________________________________ Date of Birth: ______________________________

Gender: Female / Male Handedness: Right / Left

	yes	No
I have read the information sheet		
I have received enough information about the study		
I had a chance to ask questions		
I have received satisfactory answers to my questions		
I understand that I am free to leave the study:<br>· at any time?<br>· without having to give a reason for leaving?<br>· without affecting my medical care?		
I agree that my hand movements and eye gaze be recorded		
I agree that hand and eye movements data will be shared with the research partners in Munich and Madrid.		
I agree that my hand and gaze movement data be presented on the cogwatch webpage and be made available to the general public.		
I agree that my session will be video taped		
I understand that the videos will include information that can identify me		
I agree that the data from the videos will be shared with the research partners in Munich and Madrid.		
I agree that my data from the videos be presented on the cogwatch webpage and be made available to the general public.		
If applicable, I agree that the current research will use brain-MRI and BCoS behavioural data previously collected from me by researchers at the School of Psychology		

Participant signature: ………………………………..……………………..
Name of witness: ....
Witness signature: ....
Date: .... **Participants ID: ____________________**
